# Precise spatiotemporal control of voltage-gated sodium channels by photocaged saxitoxin

**DOI:** 10.1038/s41467-021-24392-2

**Published:** 2021-07-07

**Authors:** Anna V. Elleman, Gabrielle Devienne, Christopher D. Makinson, Allison L. Haynes, John R. Huguenard, J. Du Bois

**Affiliations:** 1 Department of Chemistry, Stanford University, Stanford, CA USA; 2 Department of Neurology & Neurological Sciences, Stanford University School of Medicine, Stanford, CA USA; 3 Institute for Genomic Medicine, Columbia University Irving Medical Center, New York, NY USA; 4 Department of Neurology, Columbia University Irving Medical Center, New York, NY USA

**Keywords:** Sodium channels, Ion channels in the nervous system, Chemical tools

## Abstract

Here we report the pharmacologic blockade of voltage-gated sodium ion channels (Na_V_s) by a synthetic saxitoxin derivative affixed to a photocleavable protecting group. We demonstrate that a functionalized saxitoxin (STX-eac) enables exquisite spatiotemporal control of Na_V_s to interrupt action potentials in dissociated neurons and nerve fiber bundles. The photo-uncaged inhibitor (STX-ea) is a nanomolar potent, reversible binder of Na_V_s. We use STX-eac to reveal differential susceptibility of myelinated and unmyelinated axons in the corpus callosum to Na_V_-dependent alterations in action potential propagation, with unmyelinated axons preferentially showing reduced action potential fidelity under conditions of partial Na_V_ block. These results validate STX-eac as a high precision tool for robust photocontrol of neuronal excitability and action potential generation.

## Abstract (web summary)

Photocaged molecules have advantages in terms of temporal and spatial control compared to conventional pharmacological compounds. The authors present a synthetic saxitoxin derivative affixed to a photocleavable group for precise modulation of Na channels.

## Introduction

Small molecules bearing photocleavable protecting groups^1^ have been used extensively to manipulate biochemical processes, including protein binding interactions^2–4^, receptor activation^5^, and signaling pathway dynamics^6,7^. Photocaged molecules provide unique advantages over traditional pharmacologic agents and pro-drugs, namely precise temporal and spatial release, tunability, and reagentless deprotection. Neuroactive compounds including GABA^8^, glutamic acid^9,10^, dopamine^11^, serotonin^12^, Leu-enkephalin^13^, cAMP^14^, lipids^15^, 4-aminopyridine^16–18^, and calcium^19^ have been caged in order to study signaling mechanisms in cells, tissues, and whole organisms. Despite this impressive collection of photo-protected reagents, to our knowledge, no such compound has been designed to directly target action potential (AP) initiation in electrically excitable cells. Herein, we demonstrate that photochemical release of a caged form of saxitoxin (STX-eac) impedes AP initiation and propagation by blocking voltage-gated sodium ion channel (Na_V_) function. Experiments with dissociated neuronal cells and in tissue slice validate the utility of this unique tool compound for studies of electrical signaling.

Modern methods in neuroscience enable precise optical and chemical control of neuronal activity. Viral transduction of optogenetic and chemogenetic ion channels and receptors makes possible specific activation or inactivation of defined cell populations^20–23^. These technologies are neuromodulating (i.e., the likelihood of signaling is increased or decreased) and generally do not completely silence neuronal outputs^24,25^. Na_V_s are responsible for mediating AP initiation and propagation and are therefore an ideal target for controlling electrical transmission. Furthermore, the location, amplitude, frequency, and timing of sodium ion influx are important factors that determine electrical excitability, synaptic release, and ultimately information flow through neural systems^26,27^.

In addition to affecting AP properties, a growing body of evidence implicates Na_V_ activity in modulating dendritic currents, synaptic release, homeostasis, circuit function, and ultimately, behavior^28^. Studies of Na_V_ physiology have primarily relied on the pharmacological blockade of axon excitability using partially inactivating concentrations^29,30^ or local pressure puffing of small-molecule inhibitors^31,32^; however, these methods do not provide the fine spatial or temporal resolution required to specifically inactivate selected cells or Na_V_s in cellular compartments. The design and development of photocaged Na_V_ inhibitors for precise control of neuronal membrane excitability address these limitations.

## Results

### Synthesis of photocaged STX derivatives

**Fig. 1:**
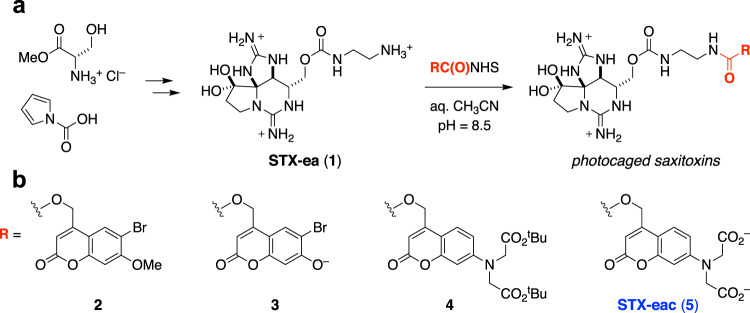
Caging STX-ea 1 with photo-protecting groups. **a** Synthesis and selective carbamoylation of STX-ea **1**. **b** Photocaged derivatives of STX-ea.

In order to achieve spatiotemporal control of Na_V_s, a photocaging group was appended to a synthetic derivative of saxitoxin (STX), a naturally occurring bis-guanidinium toxin that specifically inhibits the action of six of nine Na_V_ subtypes (e.g., IC_50_ = 1.2 nM vs. rat Na_V_ 1.2)^33^. STX targets the outer mouth of the Na_V_ pore, thereby occluding ion passage into cells. The location of the STX-binding site enables extracellular application, and block by this small-molecule toxin is entirely reversible. Modification of the N21 carbamate of STX affords saxitoxin ethylamine (STX-ea **1**)^34^, a toxin derivative of similar potency to that of the natural product (IC_50_ = 14.4 nM vs. rat Na_V_1.2). Like STX, STX-ea **1** is a reversible pore blocker that exerts no effect on Na_V_ voltage-dependence of activation or inactivation (Supplementary Fig. 1a–c). Different prosthetic groups, including photolabile carbamate derivatives, can be attached to the primary amine in STX-ea through selective acylation reactions. This tactic allows for the introduction of the photocage in the final step of the synthetic sequence and is considerably more efficient than the protection of either or both of the guanidinium groups. We exploited this chemistry to access four coumarin-derived photocaged STXs, including STX-eac **5** (Fig. 1).

Coumarin photo-protecting groups are well-established and display excellent one- and two-photon uncaging efficiencies^35,36^. In addition, the byproducts of uncaging are generally inert to biological tissue^37^. Based on models of STX binding to the channel pore and structure–activity studies of toxin binding^38,39^, we speculated that attachment of a sterically large, anionic photo-protecting group to STX-ea **1** would significantly destabilize toxin binding. The outer mouth of the channel funnels to the selectivity filter (the narrowest region of the ion permeation pathway), and STX is tightly lodged in this receptor site^38^. In addition, multiple aspartate and glutamate groups surround the toxin. Accordingly, conjugation of two different anionically charged coumarin protecting groups, 6-bromo-7-hydroxy-4-(hydroxymethyl)coumarin (pK_a_ = 6.2, λ_max_ ~375 nm)^40–42^ and 7-bis(carboxymethyl)-4-(hydroxymethyl)coumarin (λ_max_ ~380 nm)^35,43^, to **1** were prioritized, yielding compounds **3** and **5**, respectively. Two additional coumarin derivatives, **2** and **4**, were prepared for comparative purposes and to better understand the requisite structural modifications that destabilize the binding of the photocaged toxin to Na_V_^44,45^.

### Validation of photocaged STX derivatives

**Fig. 2:**
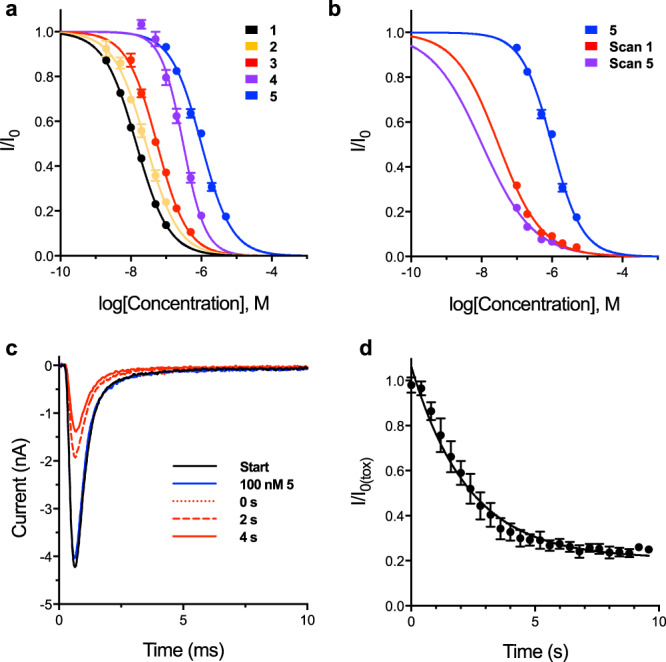
Photochemical release of STX-ea 1 results in a block of Na_V_1.2 CHO. **a** Electrophysiological characterization of photocaged STXs against Na_V_1.2 CHO. IC_50_s, Hill coefficients: **1** = 14.4 ± 0.3 nM, –0.94 ± 0.02; **2** = 27.0 ± 1.4 nM, –0.94 ± 0.05; **3** = 55.5 ± 2.1 nM, –1.01 ± 0.04; **4** = 307.5 ± 16.6 nM, –1.37 ± 0.09; **5** = 1003.8 ± 42.2 nM, –1.00 ± 0.04. Data represent mean ± s.e.m. (for compound **1** [2, 5, 10 nM], *n* = 6, [20, 50, 100 nM], *n* = 7; **2**, *n* = 3; **3**, *n* = 6; **4**, *n* = 6; **5**, *n* = 5). **b** Electrophysiological characterization and laser uncaging of **5** against Na_V_1.2 CHO. Initial IC_50_ in blue; apparent IC_50_ following laser scan 1 in red (30.4 ±  3.6 nM) and after laser scan 5 in purple (10.4 ± 2.0 nM). Data represent mean ± s.e.m. (*n* = 5). **c** Representative trace depicting uncaging of 100 nM **5** against Na_V_1.2 CHO. Traces collected in the order: Start, 100 nM **5**, 0 s, 2 s, 4 s. Laser applied immediately prior to 10 ms, 0 mV voltage step (trace 0 s). **d** Time course of uncaging of 100 nM **5** against Na_V_1.2 CHO pulsed from –100 mV to 0 mV at 2.5 Hz. The laser was applied at *t* = 0 s. Data were subjected to exponential regression yielding τ = 2.3 ± 0.2 s (half-life 1.6 ± 0.1 s), *R*^2^ = 0.9056. Data represent mean ± s.e.m. (*n* = 4). *n* = number of biologically independent cells.

All photo-protected STXs were evaluated for binding affinity by whole-cell electrophysiology against Chinese hamster ovary (CHO) cells stably expressing rNa_V_1.2. Compounds **2**–**5** were up to 70 times less potent than STX-ea **1**, with **5** displaying the highest IC_50_ value of >1 µM (Fig. 2a). As predicted, photocaged STXs bearing anionic groups are less potent than their uncharged counterparts (IC_50_: **2** = 27 nM vs. **4** = 56 nM; **3** = 308 nM vs **5** = 1.004 µM). Similarly, increasing steric bulk also impairs toxin binding (*cf*. **4** and **5** vs **2** and **3**).

In order to evaluate the uncaging efficiencies of photocaged STXs, voltage-clamped Na_V_1.2 CHO cells bathed in reagent were pulsed with 355 nm light (5 × 5 ms) while monitoring changes in channel block (Fig. 2b and Supplementary Fig. 2). STX-eac **5** exhibited the highest uncaging efficiency, as measured by comparing the change in IC_50_ of the caged compound against the apparent IC_50_ following light exposure. For **5**, 5 × 5 ms of light exposure was highly effective at removing the coumarin group (**5** = 1.004 µM vs. 10 nM post uncaging). By comparison, compounds **2**, **3**, and **4** displayed ≤3-fold change in IC_50_ under these same conditions. Control experiments on cells treated with STX-ea **1** and subjected to UV light showed no laser-dependent changes in Na_V_ activity (Supplementary Fig. 1d–f).

Following light exposure, all coumarin-modified STXs uncage and achieve maximal block of Na_V_1.2 within ~4 s. Time-course experiments with the most promising photocaged toxin, **5**, revealed a time constant for exponential current decay of 2.3 ± 0.2 s (i.e., half-maximal block achieved within 1.6 ± 0.1 s; Fig. 2c, d). The rapid time constant for block by the uncaged product, STX-ea **1**, is consistent with the measured association constant of this inhibitor (k_on_ = 11.0 ± 0.7 × 10^6^ M^–1^ s^–1^), which is similar to STX itself (Supplementary Fig. 1g, h). As noted for STX, **1** rapidly dissociates from the channel pore upon perfusion of cells with buffer solution (k_off_ = 3.8 ± 0.1 × 10^2^ s^−1^).

### Interruption of action potential (AP) trains in primary neurons

**Fig. 3:**
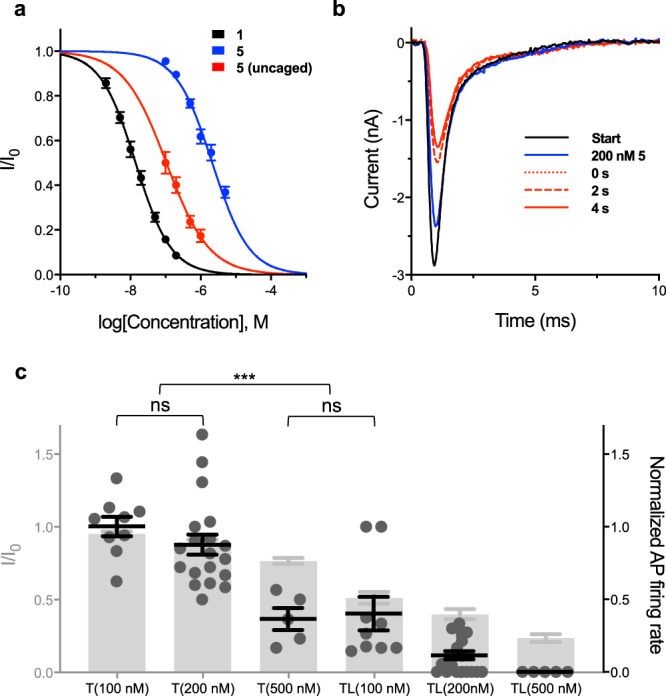
Uncaging of STX-eac 5 results in fast, concentration-dependent Na_V_ block and inhibition of APs. **a** Electrophysiological characterization of photocaged STXs against hippocampal neurons DIV 6–8. IC_50_s, Hill Coefficients: **1** = 14.1 ± 0.8 nM, –0.86 ± 0.04; STX-eac **5** = 2148.4 ± 209.9 nM, –0.83 ± 0.07. Apparent IC_50_, Hill Coefficient: **5** (uncaged) = 106.0 ± 17.4 nM, –0.71 ± 0.11. Data represent mean ± s.e.m. (for compound **1** [2 nM], *n* = 4; [5, 10, 20, 50, 100 nM], *n* = 5. For compound **5** [100 nM], *n* = 12; [200 nM], *n* = 10; [500 nM], *n* = 13; [1 µM], *n* = 12; [2 µM, 5 µM], *n* = 4. For compound **5** (uncaged) [100 nM], *n* = 4; [200 nM], *n* = 6; [500 nM], *n* = 5; [1 µM], *n* = 4.). **b** Representative trace depicting uncaging of 200 nM **5** against hippocampal neurons DIV 6–8. Traces collected in the order: Start, 200 nM **5**, 0 s, 2 s, 4 s. Laser applied immediately prior to 10 ms, 0 mV voltage step (trace 0 s). **c** Comparison between current-clamp (DIV 9–13, right axis, black) and voltage-clamp (DIV 6–8, left axis, gray) data collected pre- and post-uncaging at various concentrations of **5**. T, toxin applied; TL, toxin and laser applied. Statistics calculated for current-clamp data. ns *P* > 0.5, ***P < 0.001, one-way ANOVA with Tukey’s correction, mean ± s.e.m. T(100 nM) vs. T(200 nM), *P* = 0.7727; T(100 nM) vs. T(500 nM), *P* = 1.34 × 10^–4^; T(200 nM) vs. T(500 nM), *P* = 8.46 × 10^–4^; T(500 nM) vs. TL(100 nM), *P* = 0.9997. (For T(100 nM), *n* = 9; T(200 nM), *n* = 19; T(500 nM), *n* = 5; TL(100 nM), *n* = 9; TL(200 nM), *n* = 19; TL(500 nM), *n* = 5.) *n* = number of biologically independent cells.

To assess the utility of photocaged STX-ea to block APs, STX-eac **5** was evaluated against cultured rat embryonic hippocampal neurons (E18). In order to preserve cell health, laser excitation at 355 nm was limited to a 1 × 5 ms pulse. Against Na_V_s in primary neurons, the IC_50_ value for **5** was determined to be 2.15 ± 0.21 µM. The twofold larger IC_50_ value compared to that obtained against Na_V_1.2 in CHO cells may be due to the presence of multiple STX-sensitive Na_V_ isoforms (1.1–1.3, 1.6)^46^ against which the potency of **5** is slightly varied (Supplementary Fig. 3) or from the co-expression of β-auxiliary proteins (and/or other accessory proteins) in hippocampal neurons^27^. Analogous to experiments using CHO cells, the uncaging efficiency of **5** is high, as the apparent IC_50_ drops from 2.1 µM to 106 nM following a single 5 ms laser pulse (Fig. 3a, b). Maximum channel inhibition is achieved after 2 s with a time constant of ~1 s (Supplementary Fig. 4a).

**Fig. 4:**
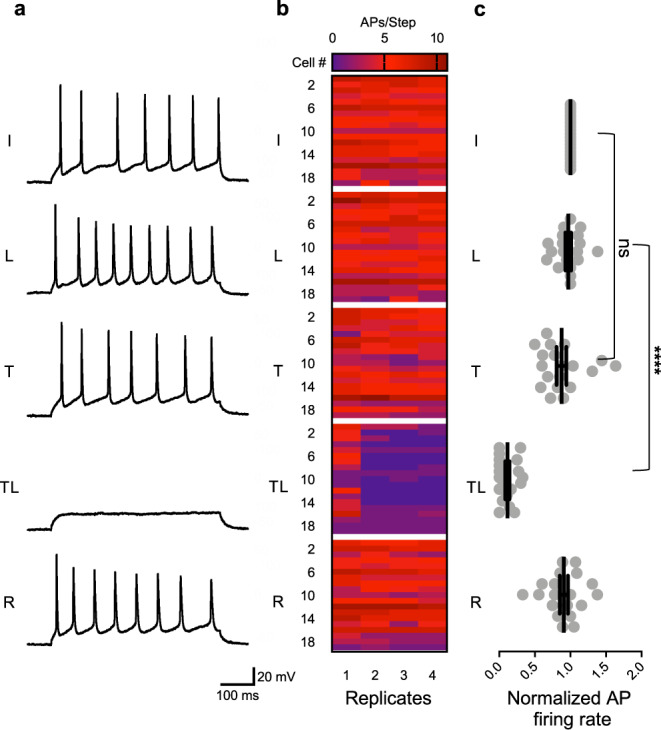
Uncaging of 200 nM STX-eac 5 shows reversible inhibition of AP firing. **a** Representative traces depicting initial (I), laser applied (L), 200 nM toxin **5** applied (T), 200 nM toxin **5** and laser applied (TL), and recovered (R) after wash-off AP trains evoked by 500 ms, 50–150 pA current injections into hippocampal neurons DIV 9–13. Data taken from replicate current step 2 vis-à-vis (**b**). **b** Heatmap summary of data described in **a** color-coded by the number of action potentials per step (four replicate current steps at 0.25 Hz per condition, *n* = 19). **c** Equilibrated normalized action potential firing rate (i.e., over current steps 2–4) pre- and post-laser induced uncaging of **5**. ns *P* > 0.5, ****P < 0.0001, one-way ANOVA with Tukey’s correction, mean ± s.e.m. I vs. L, *P* = 0.9503; I vs. T, *P* = 0.4131; L vs. T, *P* = 0.7437; I vs. TL, *P* = 2.7 × 10^–14^; L vs. TL, *P* = 9.69 × 10^–12^; T vs. TL, *P* = 2.38 × 10^–8^. (*n* = 19). *n* = number of biologically independent cells.

Given the effectiveness of **5** for inhibiting Na_V_ activity in hippocampal neurons, we next tested the ability of this reagent to impede action potential (AP) trains. Current-clamped dissociated neurons were bathed in varying concentrations of **5** to monitor the effects on AP firing frequency (Supplementary Figs. 5–7). Notably, at 100–200 nM concentration, **5** has no influence on AP firing rate, despite blocking ~5–10% of the total population of Na_V_s (Fig. 3c). Subsequent uncaging, particularly in experiments using 200 nM **5**, dramatically reduced spike frequency, on a timescale consistent with voltage-clamp recordings (τ ~ 1.1 s; Supplementary Fig. 4b). Analysis of both voltage- and current-clamp data from hippocampal neurons indicates that ≥75% channel block is needed to completely eliminate AP trains (Fig. 3c), consistent with previous reports^47,48^. Notably, blocking ~10–25% of Na_V_s resulted in the steepest decline in AP firing rate. Thus, it is possible to vary the concentration of **5**, laser power, and/or exposure time to alter AP frequency. For example, 200 nM **5** subjected to a single 5ms laser pulse produced a six-fold increase in Na_V_ block—from 10 to 60% inhibition pre- to post uncaging—the difference between complete maintenance of AP firing and reduction to 11.6 ± 2.9% of initial frequency, with over 40% of cells incapable of initiating a single action potential (Fig. 4a, b). AP block extends upward of 15 s, and is completely reversible upon perfusing cells with buffer (Fig. 4c and Supplementary Figs. 4–7). These data demonstrate that uncaging of **5** functions as a molecular circuit breaker to rapidly block trains of APs.

### Effect of STX-eac in a corpus callosum slice preparation

**Fig. 5:**
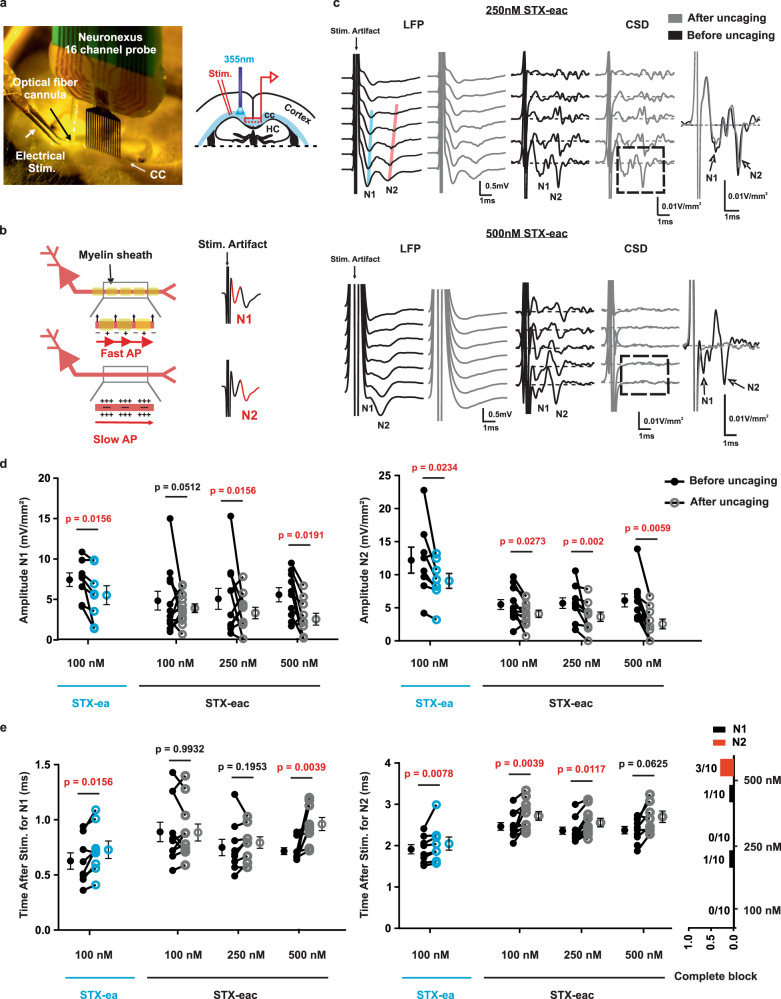
Uncaging STX-eac 5 differentially affects myelinated vs. unmyelinated callosal fiber transmission. **a** Experimental design of corpus callosum preparation. On the left, a representative picture of an experiment. On the right, a corresponding schematic representation, HC stands for the hippocampus; cc for corpus callosum. **b** Schematic representation of callosal N1 and N2 features. N1 is the fastest component and corresponds mainly to myelinated axon fibers. N2 is slower and corresponds mainly to unmyelinated axon fibers. **c** Example of LFP and CSD signals for two different concentrations of **5**, 250 nM (upper panel) and 500 nM (lower panel). Black traces represent signals before uncaging, gray traces represent signals after uncaging by 500ms UV pulse light delivery. Only the first seven channels closest to the stimulating electrode are represented. The expanded timescale of the first CSD channel is represented on the right. For 250 nM **5**, a reduction of amplitude and a delay of the peak time can be observed after uncaging for N2. Only a reduction of amplitude can be observed for N1; peak time was not affected. For 500 nM **5**, a loss of N1 and N2 signals can be observed after light delivery. **d** Scatter plot of the effect of 100 nM bath application of **1** in blue and of the effect of uncaging of 100, 250, and 500 nM **5** on N1 (left graph) and N2 (right graph) amplitude. **e** Scatter plot of the effect 100 nM bath application of **1** in blue and of uncaging of 100, 250, and 500 nM **5** on the N1 (left) and N2 (middle) peak time. Histograms (on the right) represent the incidence of complete block for N1 (black) and N2 (red) for the three different concentrations of **5**, 100, 250, and 500 nM. Increases in peak timing were observed with 100 nM **1** and 500 nM **5** for N1 and with 100 nM **1**, 100 nM and 250 nM **5** for N2. Notice that 30% of the slices have a complete block of N2 at 500 nM, obscuring any potential difference in latency. **d**, **e** Two-tailed Wilcoxon test (*n* = 8, 7, 5, and 8, respectively, for 100 nM **1**, 100 nM, 250 nM, and 500 nM **5**). *P* values less than 0.05 are highlighted in red. Each pair of connected dots represents the results of a single slice before (filled circle) and after (open circle) light application. For each condition mean ± s.e.m. are represented on each side of the connected dots.

To evaluate the functional effect of STX-eac **5** on axons, we performed electrophysiological analyses of compound action potentials (CAPs) evoked in the corpus callosum (Fig. 5a). CAPs have been previously characterized in the mouse corpus callosum as a biphasic response with an early component (1–2 ms latency) representing mainly fast and large myelinated axons (N1) and a later component (3–6ms latency) representing slower unmyelinated axons (N2)^49–51^ (Fig. 5b). Previous studies of callosal signaling have been conducted at room temperature to slow down transmission speed in order to improve N1 detection^51^; our experiments were performed at 34 °C to more closely match physiological conditions. To improve detection of N1 and N2, we estimated current-source density (CSD, see “Methods” section), which represents the inflow of Na^+^ into axons during the compound action potential. The first and second anti-peaks of the extracted CSD were used to determine the amplitude and timing of N1 and N2 action potential signals. This method of analysis was more accurate than measuring peak fiber volley in the local field potential (LFP) itself, as the latter was partially obscured by the electrical stimulus artifact. Examples of LFP and corresponding CSD traces are shown for 250 nM and 500 nM concentrations of **5** (Fig. 5c). The amplitudes and timing of N1 and N2 peaks were determined for channels closest to the stimulating electrode.

In order to uncage **5**, an optical fiber was placed close to the stimulating electrode, in contact with the upper surface of the slice, to deliver UV light (Fig. 5a). For each slice, we determined that exposure to UV light itself had a negligible effect on electrophysiological responses (Supplementary Fig. 8). Quantitative analysis of peak amplitude and frequency revealed that the two CAP components, N1 and N2, are differentially affected by uncaging **5**. By design, **5** was focally uncaged along the path of the callosal fibers between the site of initiation in the lateral corpus callosum, and propagation was measured along the midline. Uncaging of 250 nM and 500 nM **5** produced N1 CAP amplitude decreases of 23% and 37%, respectively (Supplementary Table 1). N2 was more sensitive to uncaged **5**, with N2 peak sink amplitude reduced at all three concentrations of this compound–25% at 100 nM, 36% at 250 nM, and 58% at 500 nM (Fig. 5d and Supplementary Table 1). These results indicate that transmission in unmyelinated callosal axons is more susceptible to partial sodium channel block than in myelinated fibers.

Our protocol for uncaging of **5** near the stimulation site revealed that callosal transmission speed (away from the uncaging location) along the midline is relatively insensitive to Na_V_ block; however, the arrival time of the CAP at the recording electrode is delayed, especially for the N2 component. Peak latency for N1 was only affected after uncaging the highest concentration of **5**, 500 nM, slowing by 0.28 ms. These results contrast rather starkly recordings of N2, which show a delayed peak response of ~0.2 ms with 100 nM or 250 nM **5**, and suggest a slowed CAP conduction that is restricted to the portion of axons near the site of uncaging. The effect of uncaging 500 nM **5** on N2 CAP peak onset could not be reliably determined, as the signal is undetectable in 30% of these measurements (Fig. 5c, e and Supplementary Table 1). Although signal arrival time of the CAP at the callosal midline was reduced by uncaged **5**, callosal fiber velocity, as estimated by the peak latency recorded along the midline of the callosum, was largely unaffected (Supplementary Fig. 9 and Supplementary Table 1).

Our findings demonstrate that focally uncaging **5** enables studies of action potential initiation and propagation, and that this tool compound could be particularly useful for modulating CAPs in unmyelinated axons. We wondered, however, if the differential effect of focally uncaged **5** on N1 vs. N2 might be due to an underlying sensitivity in the Na_V_s responsible for action potential initiation or propagation in myelinated vs. unmyelinated fibers. To address this possibility, we tested the sensitivity of N1 and N2 to global, steady-state (i.e., bath) application of the active parent compound, STX-ea **1**. Quantitative analysis of peak amplitude (Fig. 5d) and onset (Fig. 5e) revealed that N1 and N2 are similarly affected by bath application of 100 nM **1**, which decreases N1 and N2 CAP amplitudes by 26% (Supplementary Table 2). In addition, bath application of **1** consistently affects callosal propagation speed, with a delayed peak response at the nearest electrode of both N1 and N2 CAP by ~0.1 ms each. In contrast with targeted sodium channel inhibition by photo-uncaging **5**, CAP velocity is significantly slowed for both N1 and N2 with bath application of **1**: from 1.36 ± 0.16 m/s to 1.00 ± 0.08 m/s for N1, and from 0.53 ± 0.01 m/s to 0.43 ± 0.03 m/s for N2 (Supplementary Table 2).

## Discussion

STX-eac **5** is a high-precision molecular tool capable of spatiotemporal inhibition of Na_V_s to block the transmission of action potentials. Application of **5** to primary neuronal cells and tissue does not alter endogenous electrical signaling. Focal uncaging drives rapid release of STX-ea **1**, which achieves maximal Na_V_ inhibition within 2 s following laser excitation. The percent of Na_V_ block can be regulated by attenuating the laser power and duration of light exposure in addition to the concentration of **5**. We have demonstrated that **5** is well tolerated by cells for prolonged periods of time and that the effects of uncaging are fully reversible, with peak sodium currents restored upon perfusion of cells or tissue slice with buffer solution.

In cultured hippocampal neurons and in brain slice, **5** functions as a reversible molecular switch on AP generation. Due to the large potency difference between **5** (IC_50_ = 2.1 µM) and the uncaged compound, **1** (IC_50_ = 14 nM), complete block of evoked AP trains can be achieved following light exposure. As STX-sensitive Na_V_ isoforms are preponderant in CNS tissue^52^, we anticipate that STX-eac **5** will be a valuable tool for examining Na_V_ activity across a broad range of cell types. Moreover, given the choice of a coumarin photo-protecting group and the reported two-photon cross-sectional efficiency of uncaging such molecules (δ_unc_ > 1 GM at 740 nm^42^), we expect **5** to be particularly beneficial for studying subcellular populations of Na_V_s. Successful two-photon uncaging of RuBi-dopamine^11^, which has a smaller k_on_ and an equivalent k_off_^53^ as compared to STX-ea **1** and a lower two-photon cross-sectional efficiency of uncaging than coumarin derivatives, supports this conclusion. While other coumarin-caged tool compounds (e.g., GABA-coumarin) have enjoyed extensive use in two-photon experiments^54^, **5** is the only reagent capable of direct inhibition of action potential initiation. As such, STX-eac **5** offers a valuable complement to photocaged neurotransmitters^55^ that have proven indispensable for investigations of the nervous system.

Na_V_ block by uncaged STX-eac **5** is spatially precise. We have observed that photo-uncaging is focal, as optical fiber placement must be within 300 µm of the callosum to differentiate the two components of the callosal CAP, N1, and N2. Focal uncaging of **5** at a precise location along the callosal tract reduces the amplitude and delays arrival of the N2 signal but does not alter propagation speed in the region where the APs are measured (i.e., outside of the region of uncaging). Control experiments involving bath application of STX-ea **1** show that reduction in action potential amplitude and speed occurs in both N1 and N2 under these conditions. Accordingly, these results demonstrate that the effect of uncaging **5** is localized, as the measured AP propagation speed would be reduced if Na_V_ block was not restricted to the site of light application.

Our data from corpus callosum recordings indicate that electrical transmission in unmyelinated fibers is more susceptible to attenuation than in myelinated fibers. We demonstrate that the faster-conducting corpus callosum signal N1 is largely insensitive to localized delivery of STX-ea **1**, whereas the slower signal N2 from unmyelinated fiber is reduced in amplitude and timing. These findings have multiple potential explanations: first, AP conduction in unmyelinated fibers, which tend to be of smaller diameter^56^, may be more susceptible to local blockade. Modeling studies have suggested that Na_V_ activity more significantly influences membrane voltage in thinner axon fibers (i.e., unmyelinated N2) than in larger axons (i.e., myelinated N1)^57^, thus local block of even a small fraction of Na_V_s could have a larger relative effect on small, unmyelinated fibers. A second contributing factor may stem from the different mechanisms of AP propagation in the two types of fibers. The corpus callosum contains diverse populations of axons, divided into roughly two groups^58^. Larger diameter axons tend to be myelinated and more reliably transmit action potentials due to saltatory conduction between insulated points (nodes of Ranvier), each expressing a high Na_V_ density^59^. Inhibition of a small number of nodes can have a relatively minor effect on AP signaling, as saltatory propagation can apparently bypass or jump across short inactive segments^60^.

We have prepared STX-eac **5** and demonstrated the value of this high-precision tool compound for the localized block of Na_V_s in dissociated neurons and brain slice. When focally uncaged by a millisecond light pulse, the release of the potent STX derivative, STX-ea **1**, results in rapid Na_V_ inhibition and impedance of APs. In slice recordings on corpus callosum tissue, focal uncaging of **5** affords specific modulation of APs in slower-conducting, nonmyelinated fibers. Future studies using this reagent may offer insight into the role of unmyelinated fibers in callosal signaling^51,61,62^ or the contributions of such fibers in demyelinating diseases such as multiple sclerosis, among other outstanding questions. We expect the availability of **5** to complement existing technologies for optical control of axonal signaling.

## Methods

### Synthesis

#### General

All reagents were obtained commercially unless otherwise noted. Organic solutions were concentrated under reduced pressure (ca. 60 Torr) by rotary evaporation. Anhydrous CH_2_Cl_2_ and HPLC-grade CH_3_CN were obtained from commercial suppliers and used as is. *N,N*-dimethylformamide (DMF) was passed through two columns of activated alumina prior to use. Triethylamine was distilled from calcium hydride.

Product purification was accomplished using forced-flow chromatography on Silicycle ultrapure silica gel (40–63 µm). Semi-preparative high-performance liquid chromatography (HPLC) was performed on a Varian ProStar model 210. Thin-layer chromatography was performed on EM Science silica gel 60 F_254_ plates (250 µm). Visualization of the developed chromatogram was accomplished by fluorescence quenching. High-resolution mass spectra were obtained from the Vincent Coates Foundation Mass Spectrometry Laboratory at Stanford University. Samples were analyzed with LC/ESI-MS by direct injection onto a Waters Acquity UPLC and Thermo Fisher Exactive mass spectrometer scanning *m/z* 100–2000. The LC mobile phase was 100% methanol and the flow rate was 0.175 mL/min.

Saxitoxin derivatives were quantified by ^1^H NMR spectroscopy on a Varian Inova 600 MHz NMR instrument using distilled DMF as an internal standard. A relaxation delay (d1) of 20 s and an acquisition time (at) of 10 s were used for spectral acquisition. The concentration of the toxin derivative was determined by the integration of ^1^H signals corresponding to the toxin and a fixed concentration of the DMF standard.

#### Compounds

Coumarins 6-bromo-4-(hydroxymethyl)-7-methoxycoumarin (**6**), 6-bromo-7-hydroxy-4-(hydroxymethyl)coumarin (**7**), and 7-[bis(tert-butoxycarbonylmethyl)-amino]-4-(hydroxymethyl)coumarin (**8**) were prepared as described in Furuta et al.^41^, Furuta et al.^44^, and Noguchi et al.^63^, respectively. Synthesis of N21-saxitoxin ethylamine (**1**) was adapted from our previously disclosed work^34^. N-Hydroxysuccinimide carbonates and photocaged STXs were synthesized as described in the Supplementary Methods.

### Plasmids

Oocyte expression vector pLCT2-rNa_V_ 1.3 was a generous gift from Dr. A. L. Goldin (University of California, Irvine, Department of Microbiology & Molecular Genetics). The full-length cDNA encoding for the alpha subunit of rNa_V_ 1.3 was excised and inserted into a low-copy modified pcDNA3.1(+) vector^64^ by VectorBuilder (Vector ID VB190704-1006vdy). Detailed information about the vector can be retrieved at vectorbuilder.com. Mammalian expression vector pZem228 containing the cDNA coding for the alpha subunit of rat Na_V_ 1.4 was a generous gift from Dr. S. R. Levinson (University of Colorado, Department of Physiology and Biophysics). Mammalian expression vector pcDNA3.1(+) containing the cDNA coding for the alpha subunit of human Na_V_1.5 originated from Dr. T. R. Cummins (Indiana University, Department of Biology).

### Cell culture

#### Chinese hamster ovary (CHO) cells stably expressing rat Na_V_ 1.2

Stably expressing Na_V_1.2 CHO cells were generously provided by Dr. W. A. Catterall (University of Washington, Department of Pharmacology). Cells were grown on 10-cm tissue culture dishes in RPMI 1640 medium with l-glutamine (Thermo Fisher, Waltham, MA) and supplemented with 10% fetal bovine serum (ATCC, Manassas, VA), 50 U/mL penicillin–streptomycin (Thermo Fisher, Waltham, MA), and 0.2 µg/mL G418 (Sigma-Aldrich Co., St. Louis, MO). Cells were kept in a 37 °C, 5% carbon dioxide, 96% relative humidity incubator and passaged approximately every 3 days. Passaging of cells was accomplished by aspiration of media, washing with phosphate-buffered saline, treatment with 1 mL of trypsin-EDTA (0.05% trypsin, Millipore Sigma, Hayward, CA) until full dissociation of cells was observed (~5 min), and dilution with 4 mL of growth medium. Cells were routinely passaged at 1 in 20 to 1 in 10 dilution.

#### Chinese hamster ovary K1 (CHO-K1) cells transiently expressing Na_V_s

CHO-K1 cells were grown on 10-cm tissue culture dishes in F12-K medium (ATCC, Manassas, VA) and supplemented with 10% fetal bovine serum (ATCC, Manassas, VA) and 50 U/mL penicillin–streptomycin (Thermo Fisher, Waltham, MA). Cells were kept in a 37 °C, 5% carbon dioxide, 96% relative humidity incubator and passaged approximately every 3 days. Passaging of cells was accomplished by aspiration of media, washing with phosphate-buffered saline, treatment with 1 mL of trypsin-EDTA (0.05% trypsin, Millipore Sigma, Hayward, CA) until full dissociation of cells was observed (~5 min), and dilution with 4 mL of growth medium. Cells were routinely passaged at 1 in 20 to 1 in 10 dilution. Lipofectamine^TM^ LTX PLUS^TM^ Reagent was used to accomplish all transient transfections, according to the manufacturer’s instructions (Thermo Fisher, Waltham, MA).

#### Rat embryonic day 18 Sprague Dawley hippocampal neurons

Prior to dissection, 5 mm diameter, 0.15mm-thick round glass coverslips (Warner Instruments, Hamden, CT) were coated overnight with 1 mg/mL poly-d-lysine hydrobromide (PDL, molecular weight 70,000–150,000 Da, Millipore Sigma, Hayward, CA) in 0.1 M, pH 8.5 borate buffer in a 37 °C, 5% carbon dioxide, 96% relative humidity incubator.

Hippocampi were dissected from embryonic day 18 fetuses into ice-cold Hibernate E (BrainBits, LLC, Springfield, IL) as previously described^65^. Following dissection, cells were dissociated in 2 mL of trypsin-EDTA for 15 min in a 37 °C, 5% carbon dioxide, 96% relative humidity incubator. Subsequently, trypsinized cells were quenched with 10 mL of quenching medium (DMEM high glucose (Thermo Fisher, Waltham, MA) supplemented with 15% fetal bovine serum, 100 U/mL penicillin–streptomycin, and 1 mM MEM sodium pyruvate (Atlanta biologicals, Flowery Branch, GA)). The tissue was allowed to settle, the supernatant was removed, and the tissue pellet was rinsed twice more with 10 mL of quenching medium. Cells were then manually dissociated into 2 mL of plating medium (DMEM supplemented with 10% FBS, 50 U/mL penicillin–streptomycin, and 1 mM MEM sodium pyruvate) by pipetting with a fire-polished 9” borosilicate glass Pasteur pipet (Fisher Scientific, Waltham, MA).

Cells were plated onto PDL-coated 5mm glass coverslips in 35 mm tissue culture dishes containing 2 mL of plating medium at a density of 200,000 cells/dish (for voltage-clamp experiments) or 600,000 cells/dish (for current-clamp experiments). After 45 min, coverslips were transferred to new tissue culture dishes containing 2 mL of maintenance medium (neurobasal supplemented with 1× B-27 Supplement, 1× Glutamax, and 50 U/mL penicillin–streptomycin (Thermo Fisher, Waltham, MA)). Cells were fed every 3–4 days by changing 50% of the working medium.

### Single-cell electrophysiology

Data were measured using the patch-clamp technique in the whole-cell configuration with an Axon Axopatch 200B amplifier (Molecular Devices, San Jose, CA). The output of the patch-clamp amplifier was filtered with a built-in lowpass, four-pole Bessel filter having a cutoff frequency of 5 kHz for voltage-clamp recordings or 10 kHz for current-clamp recordings, and sampled at 100 kHz. Pulse stimulation and data acquisition used Molecular Devices Digidata 1322A or 1550B controlled with pCLAMP software version 10.4 or 11.1, respectively (Molecular Devices, San Jose, CA).

Borosilicate glass micropipettes (Sutter Instruments, Novato, CA) were fire-polished to a tip diameter yielding resistance of 1.3–5.5 MΩ, for Na_V_1.2 CHO cells, or 3.0–9.0 MΩ, for E18 hippocampal neurons, in the working solutions.

#### Voltage-clamp recordings

For Na_V_1.2 CHO and CHO-K1 cells, the internal solution was composed of 40 mM NaF, 1 mM EDTA, 20 mM HEPES, and 125 mM CsCl (pH 7.4 with CsOH); the external solution comprised 160 mM NaCl, 2 mM CaCl_2_, and 20 mM HEPES (pH 7.4 with CsOH). For E18, DIV 6–8 hippocampal neurons, the internal solution was composed of 114.5 mM gluconic acid, 114.5 mM CsOH, 2 mM NaCl, 8 mM CsCl, 10 mM MOPS, 4 mM EGTA, 4 mM MgATP, and 0.3 mM Na_2_GTP (pH 7.3 with CsOH, 240 mOsm with glucose), while the external solution was Hibernate E low fluorescence (BrainBits, LLC, Springfield, IL).

Currents were elicited by 10 ms step depolarizations from a holding potential (–100 mV for Na_V_1.2 CHO and CHO-K1 cells expressing Na_V_1.4, –120 mV for CHO-K1 cells expressing Na_V_1.3 or 1.5, or –80 mV for E18 hippocampal neurons) to 0 mV at a rate of 0.5 Hz (unless otherwise noted). Leak currents were subtracted using a standard P/4 protocol of the same polarity. Series resistance was compensated at 80–95% with a τ_lag_ of 20 or 35 ms for Na_V_1.2 CHO and CHO-K1 cells or E18 hippocampal neurons, respectively. All measurements were recorded at room temperature (20–25 °C). Data were normalized to control currents, plotted against toxin concentration, and analyzed using Prism 8 (GraphPad Software, LLC). Data were fit to concentration-response curves to obtain IC_50_ values and expressed as mean ± s.e.m. The number of observations (*n*) was ≥5 for all reported data unless otherwise noted. For IC_50_ measurements, cells were used if they exhibited currents >1 nA upon depolarization, and I/I_0_ was within 10% of the initial current upon toxin washout.

#### Current-clamp recordings

Data were collected on DIV 9–13 hippocampal neurons firing action potential trains with frequencies greater than 5 Hz. The internal solution was composed of 130 mM CH_3_SO_3_^–^K^+^, 8 mM NaCl, 10 mM HEPES, 10 mM Na_2_phosphocreatine, 3 mM l-ascorbic acid, 4 mM MgATP, and 0.4 mM Na_2_GTP (pH 7.4 with KOH, 305 mOsm with glucose); the external solution comprised Hibernate E low fluorescence adjusted to 310 mOsm with 40 mM NaCl.

Action potentials were elicited by four 500 ms current injections of 50–150 pA at a rate of 0.25 Hz. Series resistance was typically compensated at 90–95% with a τ_lag_ of 35 ms. All measurements were recorded at room temperature (20–25 °C). Data were analyzed using Clampfit (Molecular Devices, San Jose, CA). The number of observations (*n*) was ≥5 for all reported data.

#### UV laser (355 nm)

A pulsed 355nm UV laser beam (DPSS Lasers, Model 3501-100) was directed through a 200µm core optical fiber to a 200 µm core, 0.22 NA fiber-optic cannula (Thorlabs, Newton, NJ) to the clamped cell. Unless otherwise noted, photolysis was induced by 5 ms, 130 mW UV pulses activated immediately prior to the depolarization (or current injection) step.

### Extracellular multielectrode recordings

Extracellular recordings were obtained with neocortical coronal 400 µm-thick slices obtained from young (~P35) male and female wild-type mice from the C3HeB/FeJ-Scn8amed/J strain. Slices containing the midline crossing segments of the corpus callosum (Bregma 0.4 to –1) were saved, which represents three slices per brain. Recordings were performed in a humidified oxygenated interface chamber at 34 °C and perfused at a rate of 2 ml/min with oxygenated ACSF. The ACSF contained: 10 mM glucose, 26 mM NaHCO_3_, 2.5 mM KCl, 1.25 mM NaHPO_4_, 1 mM MgSO_4_, 2 mM CaCl_2_, and 126 mM NaCl (298 mOsm).

A linear silicon multichannel probe (16 channels, 100µm interelectrode spacing, NeuroNexus Technologies) was placed in the midline of the corpus callosum. A bipolar tungsten microelectrode (each wire, 50–100 kΩ, FHC) was positioned in the corpus callosum in one hemisphere at ~1 mm from the closest channel of the recording electrode, angled to ensure that both contacts were within the boundaries of the callosum. Both electrodes were initially lowered to ~200 µm slice depth, and fine adjustments were made to optimize the signal amplitude of the evoked compound action potential (CAP), representing the summed activity of callosal fibers^44^. Biphasic electrical stimuli (±400 µA, 0.2 ms each phase) were delivered every 10 s to elicit the callosal CAP response. This evoked response has previously been characterized by a faster component generated mainly by fast-conducting myelinated axons (N1), and a slower component reflecting mainly slower unmyelinated axons (N2)^66^.

A laser pulse of 500 ms, 40 mW, 355 nm UV (DPSS Lasers, Model 3501-100) was directed through a 200µm core optical fiber to a 0.22 NA cannula (Thorlabs, Newton, NJ) placed along the callosum between the recording and stimulating electrode to photo-uncage **5**. Ultraviolet light intensity was optimized for each type of experiment and each type of tissue. To observe an artifact-free biological effect of the uncaged **5** in 400µm thick slices containing the corpus callosum, we applied a lower, non-damaging intensity (40 mW) of UV light. Under these conditions, the duration of uncaging UV light stimulation needed to be ≥500 ms. In each set of experiments, we routinely evaluated the effects of the light stimulus in the absence of **5**, to rule out nonspecific effects. Three concentrations of **5** (100, 250, and 500 nM) were tested on callosal CAP N1 and N2 in three different mice groups (respectively, *n* = 7, *n* = 5, and *n* = 8).

The following protocol was performed on each slice: application of ACSF for 5 min, delivery of a UV light pulse, 5 min of ACSF, application of STX-eac for 10 min, delivery of a UV light pulse, 5 min of uncaged version of STX-eac, 10 min wash-off with ACSF.

For the bath application experiment, 100 nM **1** was used. After 5 min of ACSF application, callosal responses were recorded to obtain a baseline. Then, **1** was applied in the bath for 10 min and 15 callosal responses were elicited and recorded. Wash-off with ACSF was next performed over 10 min.

Evoked callosal CAP field potentials elicited by electrical stimulations were digitized at 25 kHz, and stored using an RZ5D processor multichannel workstation (Tucker-Davis Technologies). Signals were band-pass filtered between 1 Hz and 3 kHz. To obtain a more reliable index of the location, direction, and magnitude of currents underlying synchronous network activity, we derived the current-source density (CSD) from raw LFP signals^67^. Assuming a uniform extracellular resistivity, the CSD can be estimated as the second spatial derivative of the LFP. CSD peak amplitudes and times of N1 and N2 components of the closest channel to the stimulating electrode were quantified using trial-averaged responses (ten trials per condition).

### Ethics oversight

All animal care and dissection procedures were approved by the Stanford Administrative Panel on Laboratory Animal Care (APLAC).

### Reporting summary

Further information on research design is available in the Nature Research Reporting Summary linked to this article.

## Supplementary information

Supplementary Information

Reporting Summary

## Data Availability

The datasets generated in the current study are available from the corresponding authors on request. All synthetic characterization data generated during this study are included in the supplemental information files.  Source data are provided with this paper.

## Source data

Source Data

## Code availability

For callosal experiment analysis, data were exported from OpenDeveloper (Tucker-Davis Technologies, Inc) to MATLAB using the provided TDT2mat.m code. A custom MATLAB code was then used to obtain CSD measurements from LFP data. The formula is as follows: csd = –diff(diff(data,1,chan),1,chan)./sp2 with data = LFP data obtained from the TDT2mat.m extraction, chan = 2, sp2 (probe spacing) = 0.1 (i.e., 100 µm).
