# Author Correction: Precise spatiotemporal control of voltage-gated sodium channels by photocaged saxitoxin

**DOI:** 10.1038/s41467-022-30054-8

**Published:** 2022-04-21

**Authors:** Anna V. Elleman, Gabrielle Devienne, Christopher D. Makinson, Allison L. Haynes, John R. Huguenard, J. Du Bois

**Affiliations:** 1 Department of Chemistry, Stanford University, Stanford, CA USA; 2 Department of Neurology & Neurological Sciences, Stanford University School of Medicine, Stanford, CA USA; 3 Institute for Genomic Medicine, Columbia University Irving Medical Center, New York, NY USA; 4 Department of Neurology, Columbia University Irving Medical Center, New York, NY USA

**Keywords:** Sodium channels, Ion channels in the nervous system, Chemical tools

Correction to: *Nature Communications* 10.1038/s41467-021-24392-2, published online 7 July 2021.

In this article the grant number R01 GM117263 relating to NIH for J. Du Bois was inadvertently omitted. The original article has been corrected.

