# Supplementary material for: Precise spatiotemporal control of voltage-gated sodium channels by photocaged saxitoxin
Source: Nat Commun. 2021 Jul 7;12:4171. doi: 10.1038/s41467-021-24392-2 (PMC8263607; doi:10.1038/s41467-021-24392-2)
Supplement: Supplementary file 1 — Supplementary Information [file 41467_2021_24392_MOESM1_ESM.pdf]

Supplementary Figures:

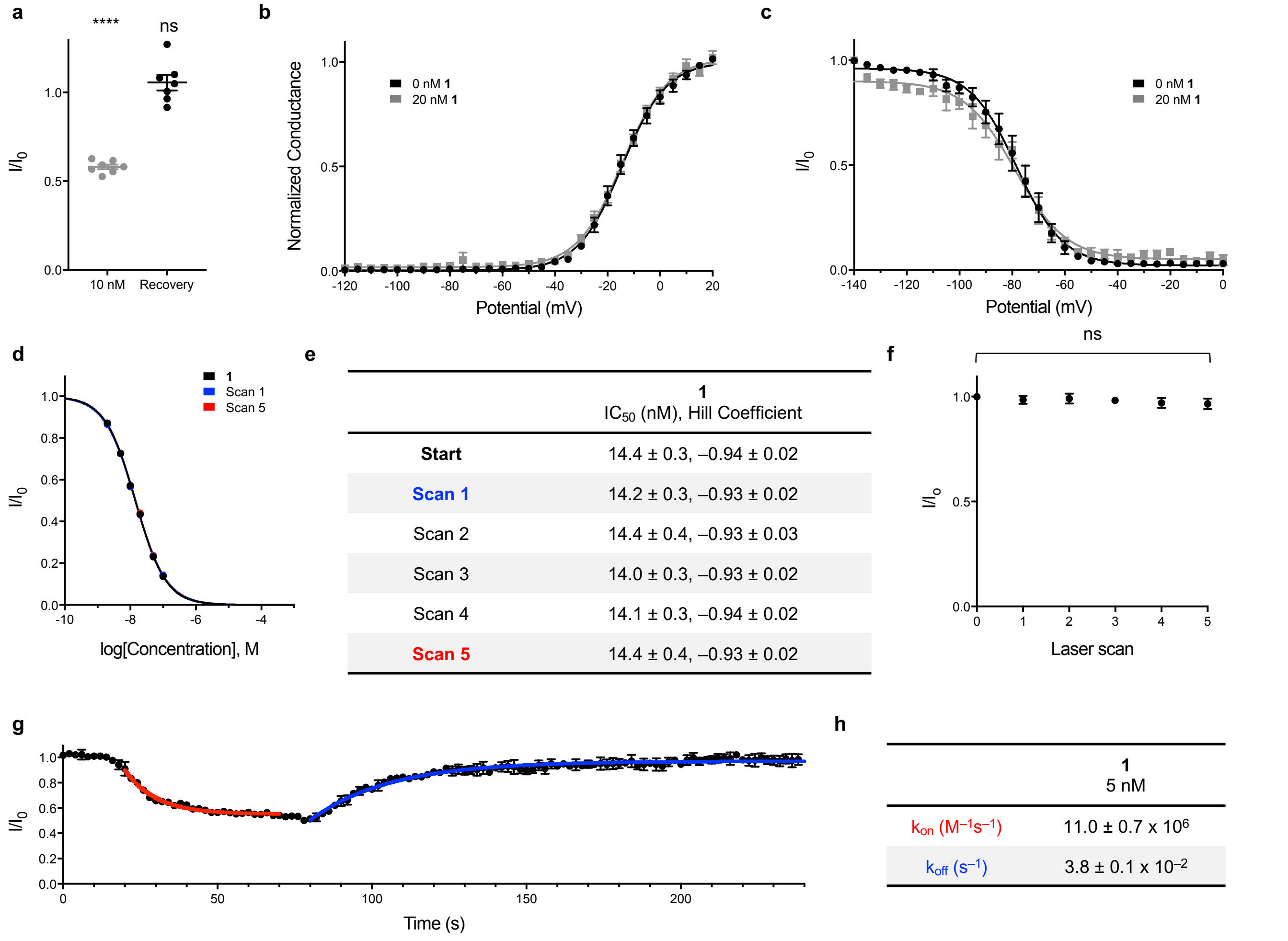

	1<br>IC_{50} (nM), Hill Coefficient
**Start**	14.4 ± 0.3, –0.94 ± 0.02
**Scan 1**	14.2 ± 0.3, –0.93 ± 0.02
Scan 2	14.4 ± 0.4, –0.93 ± 0.03
Scan 3	14.0 ± 0.3, –0.93 ± 0.02
Scan 4	14.1 ± 0.3, –0.94 ± 0.02
**Scan 5**	14.4 ± 0.4, –0.93 ± 0.02

	1<br>5 nM
k_{on} ($M^{-1}s^{-1}$)	11.0 ± 0.7 x 10^{6}
k_{off} (s^{-1})	3.8 ± 0.1 x 10^{-2}

Supplementary Figure 1. **Laser application does not alter the IC_{50} of reversible pore blocker 1 or peak current produced by Na_Vs. a** Application of 10 nM STX-ea **1** (grey) to Na_V1.2 CHO produces channel block. Wash off with 3 mL of external solution (black) yields full current recovery. ns p>0.05, ****p<0.0001, two-tailed paired t-test, mean ± s.e.m. 10 nM, p = 6.5 x 10^{-8}; Recovery, p = 0.2485. (n = 7). **b** Voltage-dependence of activation before (black) and after (grey) application of 20 nM STX-ea **1**. Current was evoked by 150 ms steps from –120 mV to 50 mV in 5 mV increments. Data represent mean ± s.e.m. (n = 5) and were fit to Boltzman sigmoidal functions. $V_{1/2}$ (Na_V1.2 CHO) = –14.4 ± 0.5 mV; $V_{1/2}$ (Na_V1.2 CHO with 20 nM STX-ea **1**) = –14.3 ± 0.5 mV. **c** Voltage-dependence of inactivation before (black) and after (grey) application of 20 nM STX-ea **1**. Current was evoked by a pre-pulse of –140 mV to 0 mV in 5 mV increments, followed by a 50 ms test pulse from –140 mV to 0 mV. Data represent mean ± s.e.m. (n = 5) and were fit to Boltzman sigmoidal functions. $V_{1/2}$ (Na_V1.2 CHO) = –78.3 ± 0.7 mV; $V_{1/2}$ (Na_V1.2 CHO with 20 nM STX-ea **1**) = –78.8 ± 0.9 mV. **d** Electrophysiological characterization of STX-ea **1** against Na_V1.2 CHO. Initial IC_{50} in black, following laser scan 1 in blue, and after laser scan 5 in red. Data represent mean ± s.e.m. (For [2, 5, 10 nM], n = 6; [20, 50, 100 nM], n = 7.) **e** Table showing apparent IC_{50}s of **1** pre- and post-laser photolysis for up to five laser scans. **f** Normalized total current produced by Na_V1.2 pre- and post-application of up to five laser scans. One-way ANOVA with Tukey's correction, mean ± s.e.m, ns p>0.05 (n = 6). **g** Time course of **1** binding to and wash off from Na_V1.2 CHO. Toxin **1** (5 nM) was applied for one minute and washed out for three minutes at a constant perfusion rate of 1 mL/min. Channels were pulsed at 2.5 Hz (abridged data shown here). Data represent mean ± s.e.m. (n = 3). Binding data (t = 20–70 s) and wash off data (t = 80–240 s) were subjected to independent exponential regressions (red and blue,

respectively), yielding $\tau(\text{on}) = 10.8 \pm 0.4$ s and $\tau(\text{off}) = 26.4 \pm 0.8$ s. **h** Association and dissociation constants were calculated for **1** against $Na_V1.2$ CHO according to the equations by Hahn and Strichartz.[1] n = number of biologically independent cells.

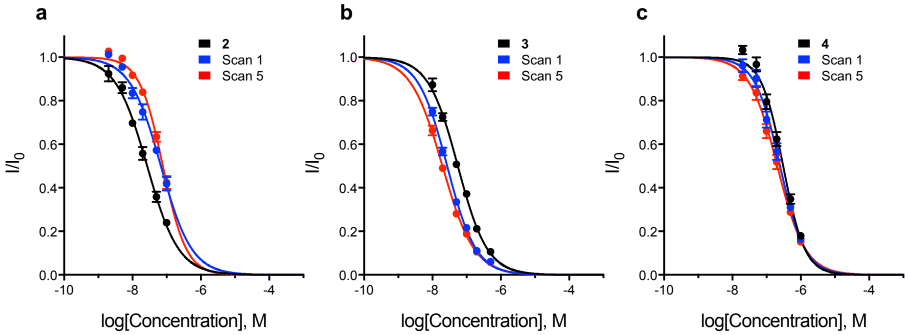

d

	**2**<br>IC_{50} (nM), Hill Coefficient	**3**<br>IC_{50} (nM), Hill Coefficient	**4**<br>IC_{50} (nM), Hill Coefficient	**5**<br>IC_{50} (nM), Hill Coefficient
**Start**	27.0 ± 1.4, –0.94 ± 0.05	55.5 ± 2.1, –1.01 ± 0.04	307.5 ± 16.6, –1.37 ± 0.09	1003.8 ± 42.2, –1.00 ± 0.04
**Scan 1**	68.1 ± 5.0, –0.96 ± 0.07	27.0 ± 0.8, –1.03 ± 0.03	248.9 ± 15.2, –1.19 ± 0.08	30.4 ± 3.6, –0.70 ± 0.04
Scan 2	77.1 ± 5.3, –1.24 ± 0.12	22.3 ± 0.9, –0.96 ± 0.04	224.1 ± 12.7, –1.12 ± 0.07	13.5 ± 2.4, –0.61 ± 0.04
Scan 3	78.6 ± 5.0, –1.31 ± 0.12	20.4 ± 0.7, –0.93 ± 0.03	223.8 ± 12.5, –1.08 ± 0.06	9.3 ± 2.2, –0.56 ± 0.04
Scan 4	86.8 ± 5.6, –1.18 ± 0.10	19.8 ± 0.6, –0.91 ± 0.03	218.9 ± 11.2, –1.09 ± 0.06	12.3 ± 1.7, –0.63 ± 0.03
**Scan 5**	77.7 ± 3.8, –1.26 ± 0.08	19.8 ± 0.7, –0.94 ± 0.03	206.9 ± 11.0, –1.06 ± 0.06	10.4 ± 2.0, –0.59 ± 0.04

Supplementary Figure 2. **Photocaged STXs 3 and 5 uncage more effectively than 2 and 4. a–c** Electrophysiological characterization of photocaged STXs **2–4** against $Na_V1.2$ CHO. Initial IC_{50} in black; apparent IC_{50} following laser scan 1 in blue and after laser scan 5 in red. **d** Table showing apparent IC_{50}s of photocaged STXs **2–5** pre- and post-laser induced uncaging for up to five laser scans. Data represent mean ± s.e.m. (For compound **2**, n = 3; **3**, n = 6; **4**, n = 6; **5**, n = 5.) n = number of biologically independent cells.

a

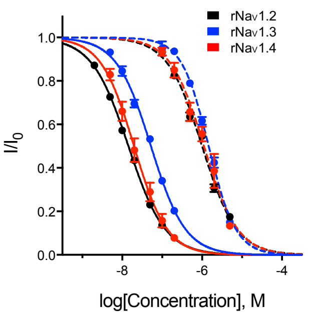

b

	1<br>IC_{50} (nM), Hill Coefficient	5<br>IC_{50} (nM), Hill Coefficient
$rNa_V1.2$	14.4 ± 0.3, –0.94 ± 0.02	1003.8 ± 42.2, –1.00 ± 0.04
$rNa_V1.3$	52.6 ± 1.9, –1.01 ± 0.04	1467.1 ± 54.5, –1.28 ± 0.06
$rNa_V1.4$	19.8 ± 1.5, –1.04 ± 0.08	1120.4 ± 99.3, –1.03 ± 0.10
$hNa_V1.5$	>2 μM	>2 μM

c

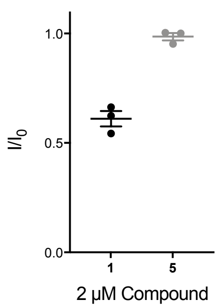

Supplementary Figure 3. **Compounds 1 and 5 exhibit similar potencies against other STX-sensitive Na_V isoforms. a** Electrophysiological characterization of **1** (solid) and **5** (dashed) against $Na_V1.2$ CHO, CHO-K1 expressing $rNa_V1.3$, and CHO-K1 expressing $rNa_V1.4$. **b** Table showing IC_{50}s of **1** and **5**. **c** Electrophysiological characterization of **1** and **5** against CHO-K1 expressing $hNa_V1.5$; data collected at 2 μM. Data represent mean ± s.e.m. (For compound **1** vs. $rNa_V1.2$, [2, 5, 10 nM], n = 6, [20, 50, 100 nM], n = 7; vs. $rNa_V1.3$, n = 3; vs. $rNa_V1.4$, n = 4; vs. $hNa_V1.5$, n = 3. For compound **5** vs. $rNa_V1.2$, n = 5; vs. $rNa_V1.3$, n = 3; vs. $rNa_V1.4$, n = 3; vs. $hNa_V1.5$, n = 3.) n = number of biologically independent cells.

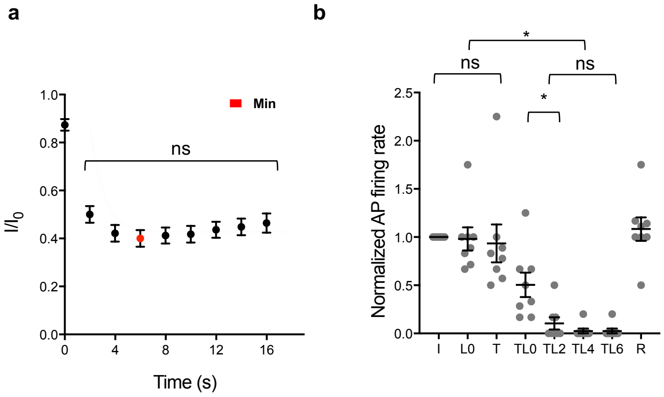

Supplementary Figure 4. **Uncaging of STX-eac 5 is functionally complete within seconds against hippocampal neurons. a** Time course of uncaging of 200 nM **5** against hippocampal neurons DIV 6–8. Laser was applied at t = 0 s. Data was subjected to exponential regression yielding $\tau = 1.0 \pm 0.3$ seconds, $R^2 = 0.7521$. (n = 6, two-way ANOVA with Tukey's correction, mean ± s.e.m.). Data from Min used to plot Figure 3. **b** Time course for uncaging of 200 nM **5** against hippocampal neurons DIV 9–13. Graph depicts normalized AP firing rates evoked by 500 ms, 50–150 pA current injections either: initially (I), 0 seconds after laser application (L0), post-toxin application (T), post-toxin application and 0, 2, 4, or 6 seconds after laser application (TL0, TL2, TL4, and TL6, respectively), and post-wash off/recovery (R). Data was subjected to exponential regression yielding $\tau = 1.1 \pm 0.8$ seconds, $R^2 = 0.5124$. ns $p>0.05$, *$p<0.05$, one-way ANOVA with Tukey's correction, mean ± s.e.m. I vs. L0, p = 1.000; I vs. T, p = 1.000; I vs. TL2, p = 2.76×10^{-5}; I vs. TL4, p = 4.38×10^{-8}; I vs. TL6, p = 4.38×10^{-8}; L0 vs. T, p = 0.9994; L0 vs. TL2, p = 7.63×10^{-5}; L0 vs. TL4, p = 0.0017; L0 vs. TL6, p = 0.0017; T vs. TL2, p = 0.0118; T vs. TL4, p = 0.0351; T vs. TL6, p = 0.0351; TL0 vs. TL2, p = 0.0436; TL2 vs. TL4, p = 0.8961; TL2 vs. TL6, p = 0.8961 (n = 8). n = number of biologically independent cells.

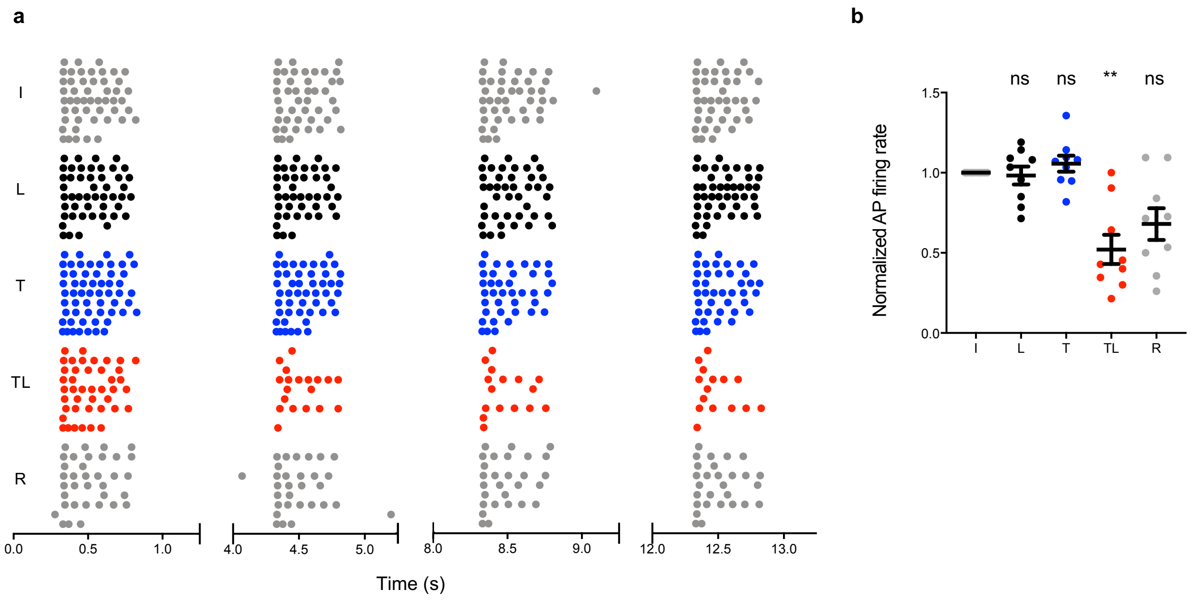

Supplementary Figure 5. **Uncaging of 100 nM STX-eac 5 with one 5 ms laser pulse is insufficient to consistently inhibit action potential trains in hippocampal neurons. a** Raster plot depicting initial (I), laser applied (L), 100 nM **5** applied (T), 100 nM STX-eac **5** and laser applied (TL), and recovered following wash off (R) action potential trains evoked by 500 ms, 50–150 pA, 0.25 Hz current injections into hippocampal neurons DIV 9–13. **b** Normalized action potential firing rates calculated for data in (**a**) ns $p>0.05$, $^{**}p<0.01$, one-way ANOVA with Tukey's correction, mean ± s.e.m. I vs. L, $p = 0.9976$; I vs. T, $p = 0.7872$; I vs. TL, $p = 0.0049$; I vs. R, $p = 0.0686$. ($n = 9$). n = number of biologically independent cells.

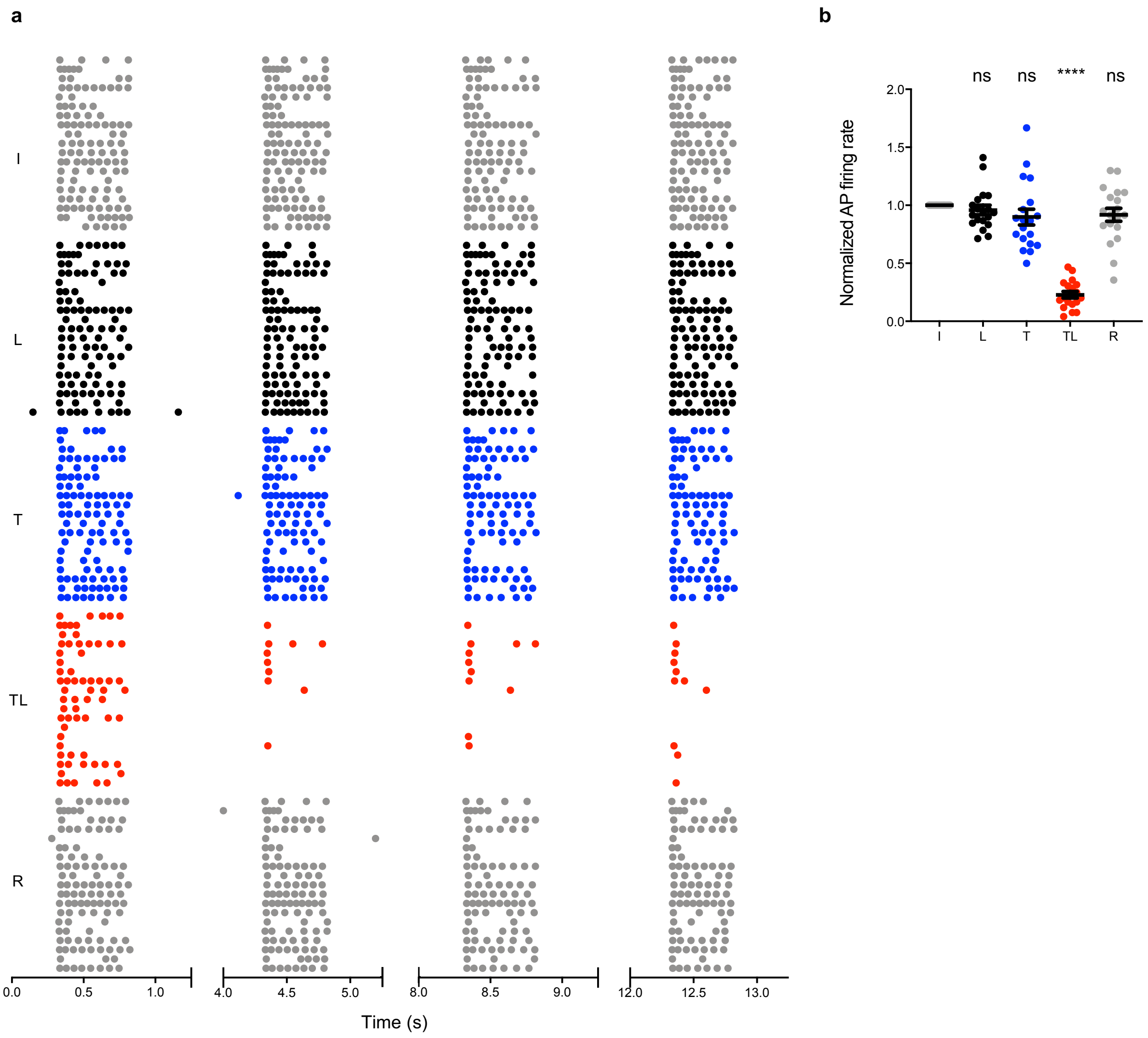

Supplementary Figure 6. **Uncaging of 200 nM STX-eac 5 with one 5 ms laser pulse is sufficient to consistently inhibit action potential trains in hippocampal neurons. a** Raster plot depicting initial (I), laser applied (L), 200 nM **5** applied (T), 200 nM STX-each **5** and laser applied (TL), and recovered following wash off (R) action potential trains evoked by 500 ms, 50–150 pA, 0.25 Hz current injections into hippocampal neurons DIV 9–13. **b** Normalized action potential firing rates calculated for data in (**a**) ns $p>0.05$, ****$p<0.0001$, one-way ANOVA with Tukey's correction, mean ± s.e.m. I vs. L, $p = 0.8629$; I vs. T, $p = 0.5908$; I vs. TL, $p = 2.9 \times 10^{-14}$; I vs. R, $p = 0.6039$. ($n = 19$). n = number of biologically independent cells.

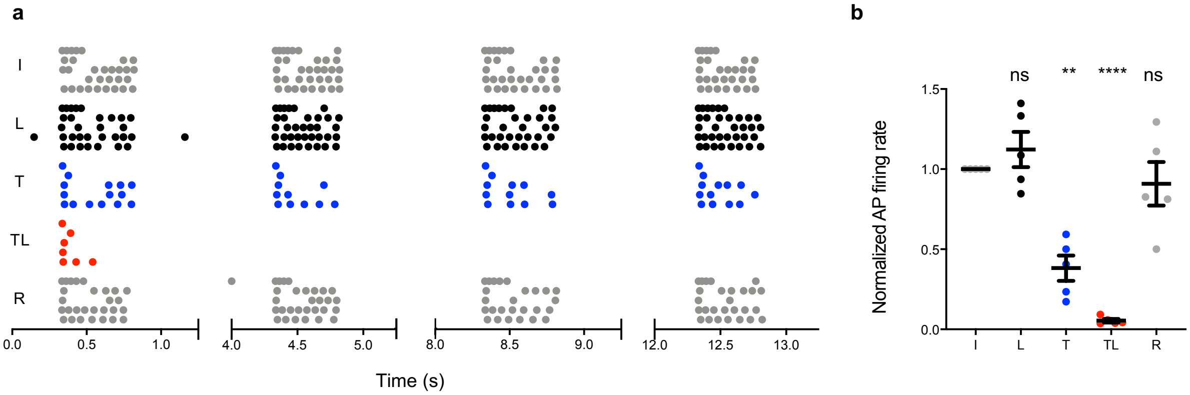

Supplementary Figure 7. **500 nM STX-eac 5 alters action potential trains absent laser-induced uncaging. a** Raster plot depicting initial (I), laser applied (L), 500 nM **5** applied (T), 500 nM STX-eac **5** and laser applied (TL), and recovered following wash off (R) action potential trains evoked by 500 ms, 50–150 pA, 0.25 Hz current injections into hippocampal neurons DIV 9–13. **b** Normalized action potential firing rates calculated for data in (**a**) ns $p>0.05$, **$p<0.01$, ****$p<0.0001$, one-way ANOVA with Tukey's correction, mean ± s.e.m. I vs. L, $p = 0.7899$; I vs. T, $p = 0.0067$; I vs. TL, $p = 3.21 \times 10^{-13}$; I vs. R, $p = 0.9537$. (n = 5). n = number of biologically independent cells.

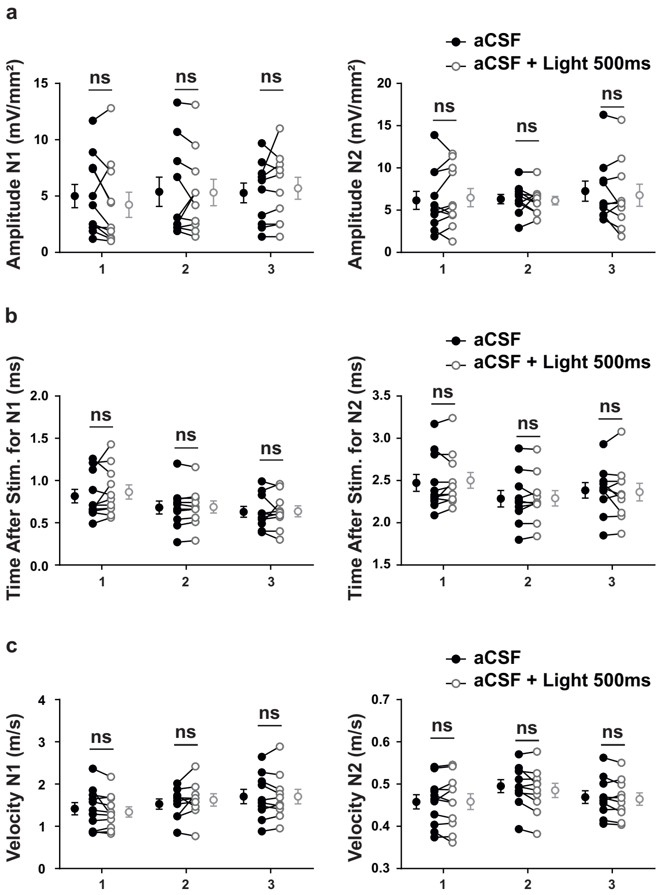

Supplementary Figure 8. **UV light has a negligible effect on corpus callosum responses. a** Effect of a 500 ms 355 nm pulse of light on the amplitude of N1 (left) and N2 (right). The effect of this UV pulse was tested in an aCSF baseline condition on each slice before the later application of STX-eac at 100 nM (1), 250 nM (2) and 500 nM (3), respectively. No significant effect of the light was observed. (For N1: 100 nM,

p = 0.1309; 250 nM, p = 0.5566; 500 nM, p = 0.5703. For N2: 100 nM, p= 0.1797; 250 nM, p = 0.5156; 500 nM, p = 0.414.) **b** Effects of a 500 ms 355 nm pulse light on the timing of peak N1 (left) and N2 (right) response prior to STX-eac **5** application. No significant effect of the light was observed. (For N1: 100 nM, p = 0.289; 250 nM, p = 0.7031; 500 nM, p = 0.886. For N2: 100 nM, p = 0.1797; 250 nM, p = 0.7422; 500 nM, p = 0.8438.) **c** Effect of a 500 ms 355 nm pulse light on the velocity of N1 (left) and N2 (right) signal prior to STX-eac **5** application. No significant effect of the light was observed. (For N1: 100 nM, p = 0.1748; 250 nM, p = 0.3008; 500 nM, p > 0.9999. For N2: 100 nM, p = 0.9658; 250 nM, p = 0.0840; 500 nM, p = 0.2324.) Two tailed Wilcoxon test, n = 7, 5, and 8 mice respectively for 100 nM, 250 nM and 500 nM **5**. ns p>0.05. Each pair of connected dots represents results of a single slice before (filled circle) and after (open circle) light application. For each condition, mean ± s.e.m. are represented on each side of the connected dots.

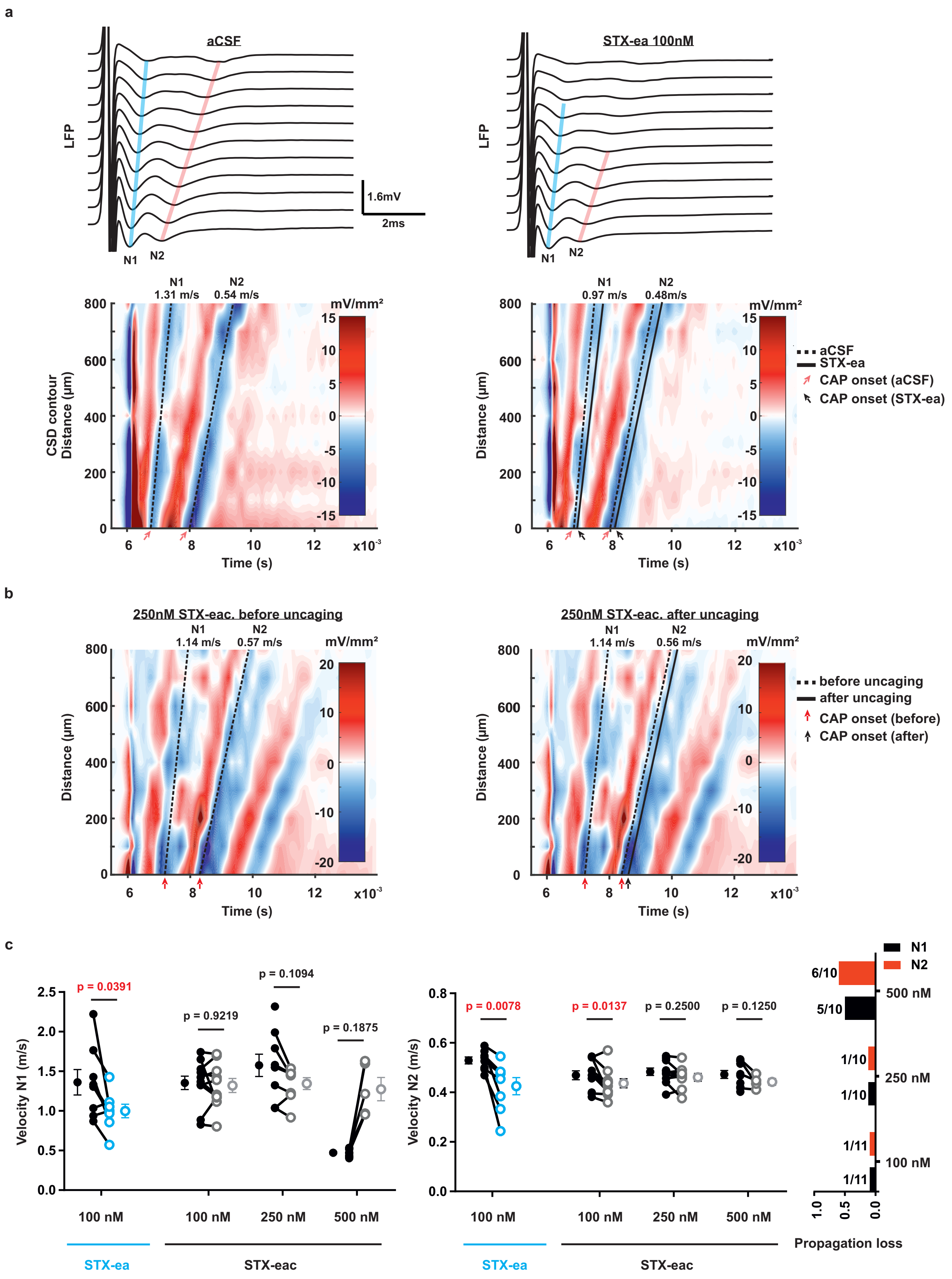
a
aCSF
STX-ea 100nM
LFP
1.6mV
2ms
N1
N2
N1 1.31 m/s
N2 0.54 m/s
N1 0.97 m/s
N2 0.48m/s
mV/mm²
CSD contour
Distance (μm)
Time (s)
x10⁻³
aCSF
STX-ea
CAP onset (aCSF)
CAP onset (STX-ea)
b
250nM STX-eac, before uncaging
250nM STX-eac, after uncaging
N1 1.14 m/s
N2 0.57 m/s
N2 0.56 m/s
before uncaging
after uncaging
CAP onset (before)
CAP onset (after)
c
p = 0.0391
p = 0.9219
p = 0.1094
p = 0.1875
p = 0.0078
p = 0.0137
p = 0.2500
p = 0.1250
Velocity N1 (m/s)
Velocity N2 (m/s)
100 nM
250 nM
500 nM
STX-ea
STX-eac
N1
N2
6/10
5/10
1/10
1/11
Propagation loss

Supplementary Figure 9. **Differential effect of 100 nM STX-ea 1 bath application and uncaged STX-eac 5 on callosal signal propagation. a** Example of LFP signals and CSD contour plot for 100 nM **1** applied in bath. The left panel represents the aCSF baseline condition. The right panel represents signals after 10 minutes of application of **1** in bath. For both conditions, 10 recordings are averaged. Only the first 11 channels closest to the stimulating electrode are represented. Propagation of N1 and N2 signals across channels is represented respectively in blue and red. In the LFP signal, a reduction of propagation of both N1 and N2 (blue and red traces are shorter) is observed in the 100 nM **1** condition. In the CSD contour plot, both reduction in propagation and delayed onset can be seen. Indeed, after **1** (right, solid lines), there is a shift in both onset and propagation speed compared to control (dashed lines). **b** Representative CSD contour plots for 100 nM **5** before and after uncaging. The left panel represents uncaged baseline condition. The right panel represents signals after exposure to 500 ms of 355 nM light. For both conditions, 10 recordings are averaged. Only the first 11 channels closest to the stimulating electrode are represented. Reduction in onset, but not propagation speed is observed for N2, while N1 is apparently unaffected. **c** Scatter plot of the effect of 100 nM bath application of **1** in blue and of uncaging of 100 nM, 250 nM and 500 nM **5** on the N1 (left) and N2 (middle) velocity. Histograms (on the right) represent the incidence of complete block for N1 (black) and N2 (red) for the three different concentrations of **5**, 100 nM, 250 nM and 500 nM. N1 and N2 velocity are both consistently decreased with bath applied **1**, while little effect was seen with uncaged **5** (except on N2 at 100 nM). Notice that half of the slices have a loss of both N1 and N2 propagation at 500 nM, obscuring any potential difference in velocity. Two tailed Wilcoxon, n = 8, 7, 5, and 8 respectively for 100 nM **1**, 100 nM, 250 nM, and 500 nM **5**. p values less than 0.05 are highlighted in red. Each pair of connected dots represents results of a single slice before (filled circle) and after (open circle) light application. For each condition mean ± s.e.m. are represented on each side of the connected dots.

Supplementary Tables:

	Amplitude (mV/mm^2)				Time after stimulation (ms)				Velocity (m/s)			
	N1		N2		N1		N2		N1		N2	
	Before	After	Before	After	Before	After	Before	After	Before	After	Before	After
100 nM	4.54 ± 1.23	4.72 ±1.34	5.50 ± 0.75	4.1 ± 0.56	0.89 ± 0.09	0.89 ± 0.08	2.47 ± 0.09	2.72 ± 0.11	1.35 ± 0.08	1.32 ± 0.09	0.47 ± 0.02	0.44 ± 0.02
	n = 11	n = 11	n = 11	n = 11	n = 11	n = 11	n = 11	n = 11	n = 11	n = 10	n = 11	n = 10
	p = 0.5117		p = 0.0273		p = 0.9932		p = 0.0039		p = 0.9219		p = 0.0137	
250 nM	4.98 ± 1.43	3.83 ± 1.19	5.70 ± 0.80	3.66 ± 0.71	0.71 ± 0.08	0.79 ± 0.05	2.36 ± 0.09	2.56 ± 0.11	1.58 ± 0.14	1.35 ± 0.08	0.48 ± 0.02	0.46 ± 0.02
	n = 10	n = 10	n = 10	n = 10	n = 10	n = 9	n = 10	n = 10	n = 9	n = 8	n = 10	n = 9
	p = 0.0156		p = 0.0020		p = 0.1953		p = 0.0117		p = 0.1094		p = 0.2500	
500 nM	5.59 ± 0.88	3.53 ± 0.69	6.12 ± 0.99	2.56 ± 0.74	0.68 ± 0.05	0.96 ± 0.06	2.40 ± 0.11	2.70 ± 0.13	1.68 ± 0.19	1.27 ± 0.15	0.47 ± 0.02	0.44 ± 0.01
	n = 10	n = 10	n = 10	n = 10	n = 10	n = 9	n = 7	n = 7	n = 9	n = 5	n = 10	n = 4
	p = 0.0191		p = 0.0059		p = 0.0039		p = 0.0625		p = 0.1875		p = 0.1250	

Supplementary Table 1. **Uncaging of STX-eac 5 in a corpus callosum slice preparation. Before**, before uncaging; **After**, after uncaging. Two-sided Wilcoxon tests were used. p values less than 0.05 are highlighted in red. n = number of analyzed slices.

	Amplitude (mV/mm^2)				Time after stimulation (ms)				Velocity (m/s)			
	N1		N2		N1		N2		N1		N2	
	aCSF	Bath	aCSF	Bath	aCSF	Bath	aCSF	Bath	aCSF	Bath	aCSF	Bath
100 nM	7.44 ± 0.85	5.53 ± 1.17	12.2 ± 1.97	9.07 ± 1.12	0.63 ± 0.07	0.73 ± 0.08	1.91 ± 0.11	2.05 ± 0.16	1.36 ± 0.16	1.00 ± 0.08	0.53 ± 0.01	0.43 ± 0.03
	n = 8	n = 8	n = 8	n = 8	n = 8	n = 8	n = 8	n = 8	n = 8	n = 8	n = 8	n = 8
	p = 0.0156		p = 0.0234		p = 0.0156		p = 0.0078		p = 0.0391		p = 0.0078	

Supplementary Table 2. **Bath application of STX-ea 1 in a corpus callosum slice preparation. aCSF**, artificial cerebrospinal fluid; **Bath**, bath application of STX-ea. Two-sided Wilcoxon tests were used. p values less than 0.05 are highlighted in red. n = number of analyzed slices.

Supplementary Methods:

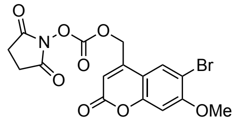

(6-Bromo-7-methoxycoumarin-4-yl)methoxycarbonyl-N-oxosuccinimide (9). To a solution of **6** (9.5 mg, 33 µmol) in 666 µL of CH_3CN was added N,N'-disuccinimidyl carbonate (17 mg, 66 µmol, 2.0 equiv) and Et_3N (4.7 µL, 33 µmol). The reaction mixture was stirred for 4 h then diluted with 5 mL of EtOAc and transferred to a separatory funnel containing 5 mL of saturated aqueous NH_4Cl. The organic layer was collected and washed successively with 1 x 5 mL of saturated aqueous NaCl and 2 x 5 mL of ½ saturated aqueous NaCl. The organic fraction was dried over Na_2SO_4, filtered, and concentrated under reduced pressure to a white powder. Purification of this material by chromatography on silica gel (gradient elution: 19:1→17:3 CH_2Cl_2/acetone) afforded **9** (2 mg, 14%) as a white powder.

TLC (9:1 CH_2Cl_2/acetone): R_f = 0.57; ^{1}H NMR (500 MHz, $CDCl_3$): δ 7.64 (s, 1H), 6.89 (s, 1H), 6.47 (s, 1H), 5.43 (s, 2H), 3.98 (s, 3H), 2.88 (s, 4H) ppm; ^{13}C NMR (125 MHz, $CDCl_3$): δ 168.3, 159.8, 159.2, 154.8, 151.4, 145.6, 127.4, 112.1, 111.0, 108.3, 100.9, 67.0, 57.0, 25.6 ppm; IR (thin film): 3093, 2923, 1817, 1791, 1732, 1608, 1413, 1274, 1204, 1080 cm^{-1}; HRMS (ESI$^+$): [MH]$^+$ calcd. for $C_{16}H_{12}BrNO_8$, 425.9819; found, 425.9808.

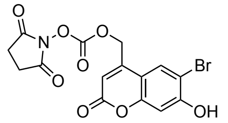

(6-Bromo-7-hydroxycoumarin-4-yl)methoxycarbonyl-N-oxosuccinimide (10). This compound was prepared in an analogous manner to **9** starting from **7** (27 mg, 100 µmol) using 2.0 equiv of triethylamine (28 µL, 200 µmol). Purification by chromatography on silica gel (gradient elution: 5:0→4:1 CH_2Cl_2/acetone) afforded **5** (25 mg, 60%) as a white powder.

TLC (9:1 CH_2Cl_2/acetone): R_f = 0.32; ^{1}H NMR (500 MHz, CD_3OD): δ 7.91 (s, 1H), 6.91 (s, 1H), 6.24 (s, 1H), 5.68 (s, 2H), 2.81 (s, 4H) ppm; ^{13}C NMR (125 MHz, CD_3OD) δ 179.2, 168.8, 167.2, 163.4, 160.4, 157.0, 138.2, 119.5, 119.4, 115.8, 112.7, 76.9, 34.9 ppm; IR (thin film): 3088, 1818, 1791, 1738, 1606, 1388, 1275, 1216, 1079 cm^{-1}; HRMS (ESI$^+$): [MH]$^+$ calcd. for $C_{15}H_{10}BrNO_8$, 411.9663; found, 411.9652.

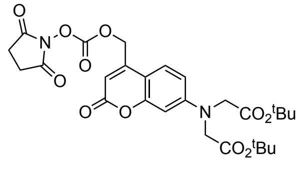

(7-[Bis(tert-butoxycarbonylmethyl)-amino]coumarin-4-yl)methoxycarbonyl-N-oxosuccinimide (11). This compound was prepared in an analogous manner to **9** starting from **8** (50 mg, 120 µmol). Purification by chromatography on silica gel (gradient elution: 7:3→1:1 hexanes/EtOAc) afforded **7** (54 mg, 81%) as a yellow oil.

TLC (1:1 hexanes/EtOAc): R_f = 0.21; ^{1}H NMR (500 MHz, $CDCl_3$): δ 7.30 (dd, *J* = 8.9, 2.2 Hz, 1H), 6.53 (dd, *J* = 8.9, 2.6 Hz, 1H), 6.47 (d, *J* = 2.6 Hz, 1H), 6.29 (s, 1H), 5.40 (s, 2H), 4.05 (s, 4H), 2.86 (s, 4H), 1.47 (s, 18H) ppm; ^{13}C NMR (125 MHz, $CDCl_3$): δ 168.9, 168.4, 161.0, 155.8, 151.6, 151.4, 146.5, 124.5, 109.4,

109.3, 107.6, 99.4, 82.7, 67.4, 54.4, 28.2, 25.5 ppm; IR (thin film): 3467, 2979, 2932, 1791, 1741, 1613, 1394, 1226, 1153 cm^{-1}; HRMS (ESI^+): $[MH]^+$ calcd. for $C_{27}H_{32}N_2O_{11}$, 561.2079; found, 561.2064.

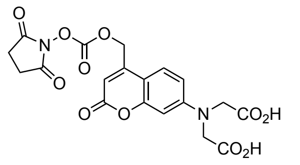

(7-[Bis(carboxymethyl)-amino]coumarin-4-yl)methoxycarbonyl-N-oxosuccinimide (12). To a solution of **11** (23 mg, 40.9 µmol) in 0.9 mL of CH_2Cl_2 was added 2.8 mL of CF_3CO_2H and 40 µL of H_2O. The reaction mixture was stirred for 30 min then concentrated under reduced pressure to afford **12** (25 mg, quantitative) as a yellow foam. This material was used in the subsequent reaction without purification.

TLC (1:1 hexanes/EtOAc): $R_f = 0.00$; ^{1}H NMR (500 MHz, d_6-DMSO): δ 7.50 (d, $J = 9.0$ Hz, 1H), 6.63 (dd, $J = 9.0$, 2.6 Hz, 1H), 6.51 (d, $J = 2.6$ Hz, 1H), 6.10 (s, 1H), 5.66 (s, 2H), 4.24 (s, 4H), 2.83 (s, 4H) ppm; ^{13}C NMR (125 MHz, d_6-DMSO): δ 171.3, 169.8, 160.13, 155.2, 151.7, 151.0, 148.3, 125.5, 109.3, 107.2, 106.4, 98.1, 67.6, 52.7, 25.4 ppm; IR (thin film) 3466, 2941, 1790, 1739, 1612, 1420, 1222 cm^{-1}; HRMS (ESI^+): $[MH]^+$ calcd. for $C_{19}H_{16}N_2O_{11}$, 449.0827; found, 449.0816.

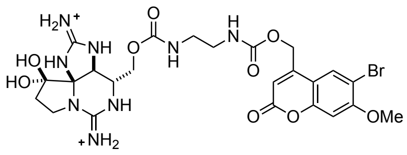

(6-Bromo-7-methoxycoumarin-4-yl)methyl N21-ethylcarbamate saxitoxin (2). To an ice-cold solution of STX-ea **1** (1.64 µmol) in 164 µL of pH 8.5 aqueous phosphate buffer (0.1 M Na_2HPO_4/Na_3PO_4) was added a solution of **9** (1.4 mg, 3.29 µmol, 2.0 equiv) in 164 µL of CH_3CN. The reaction flask was stoppered and placed in a sonication bath for 30 seconds. The flask was then wrapped in foil and the contents stirred for 5 h. Following this time, the reaction was quenched by the addition of 16.4 µL of 1.0 M aqueous CF_3CO_2H. The reaction mixture was diluted with 1.65 mL of a 9:1 10 mM aqueous CF_3CO_2H/CH_3CN solution and filtered through a Fisher 0.22 µm PTFE filter. The product was purified by reversed-phase HPLC (Silicycle SiliaChrom AQ C18, 5 µm, 10 x 250 mm column, eluting with a gradient flow of 0→40% CH_3CN in 10 mM aqueous CF_3CO_2H over 80 min, 214 nm UV detection). At a flow rate of 4 mL/min, **2** had a retention time of 47–50 min and was isolated as a white powder following lyophilization (0.48 µmol, 29%, ^{1}H NMR quantitation).

^{1}H NMR (600 MHz, D_2O): δ 7.98 (s, 1H), 7.17 (s, 1H), 6.39 (s, 1H), 5.35 (s, 2H), 4.71 (s, 1H), 4.28 (dd, $J = 11.8$, 10.0 Hz, 1H), 4.02 (s, 3H), 4.01–3.99 (m, 1H), 3.84–3.76 (m, 2H), 3.57 (dd, $J = 9.4$, 9.4, 1H), 3.40–3.24 (m, 4H), 2.43 (dd, $J = 14.2$, 7.8 Hz, 1H), 2.35 (ddd, 14.1, 9.9, 9.9 Hz, 1H) ppm; HRMS (ESI^+): $[MH]^+$ calcd. for $C_{24}H_{29}BrN_8O_9$, 653.1314; found, 653.1305.

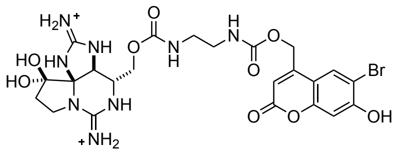

(6-Bromo-7-hydroxycoumarin-4-yl)methyl N21-ethylcarbamate saxitoxin (3). This compound was prepared in an analogous manner to **2** starting from STX-ea **1** (2.24 µmol) and **10** (1.8 mg, 4.47 µmol, 2.0 equiv). At a flow rate of 4 mL/min (gradient flow of 0→40% CH_3CN in 10 mM aqueous CF_3CO_2H over

80 min, 214 nm UV detection), **3** had a retention time of 41–42 min and was isolated as a white powder following lyophilization (1.10 µmol, 49%, ^{1}H NMR quantitation).

^{1}H NMR (600 MHz, D_2O): δ 7.95 (s, 1H), 7.03 (s, 1H), 6.36 (s, 1H), 5.33 (s, 2H), 4.70 (s, 1H), 4.28 (dd, *J* = 12.5, 9.1 Hz, 1H), 4.02–3.96 (m, 1H), 4.83–3.72 (m, 2H), 3.56 (t, *J* = 9.9, 1H), 3.41–3.21 (m, 4H), 2.46–2.39 (m, 1H), 2.38–2.30 (m, 1H) ppm; HRMS (ESI^{+}): $[MH]^{+}$ calcd. for $C_{23}H_{27}BrN_8O_9$, 639.1157; found, 639.1161.

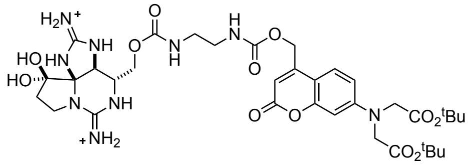

(7-[Bis(tert-butoxycarbonylmethyl)-amino]coumarin-4-yl)methyl N21-ethylcarbamate saxitoxin (4). This compound was prepared in an analogous manner to **2** starting from STX-ea **1** (1.73 µmol) and **11** (2 mg, 3.566 µmol, 2 eq). At a flow rate of 4 mL/min (gradient flow of 10→50% CH_3CN in 10 mM aqueous CF_3CO_2H over 80 min, 214 nm UV detection), **4** had a retention time of 54–56 min and was isolated as a white powder following lyophilization (0.29 µmol, 17%, ^{1}H NMR quantitation).

^{1}H NMR (600 MHz, D_2O): δ 7.60 (d, *J* = 9.0 Hz, 1H), 6.74 (dd, *J* = 9.1, 2.6 Hz, 1H), 6.66 (d, *J* = 2.6 Hz, 1H), 6.23 (s, 1H), 5.40–5.31 (m, 2H), 4.68 (s, 1H), 4.27 (s, 4H), 4.27–4.23 (m, 1H), 3.96 (dd, *J* = 11.8, 5.4 Hz, 1H), 3.77 (t, *J* = 10.0 Hz, 1H), 3.74–3.68 (m, 1H), 3.53 (dd *J* = 9.4, 9.4, 1H), 3.41–3.31 (m, 1H), 3.31–3.18 (m, 3H), 2.44–2.19 (m, 2H), 1.48 (s, 18H) ppm; HRMS (ESI^{+}): $[MH]^{+}$ calcd. for $C_{35}H_{49}N_9O_{12}$, 788.3574; found, 788.3560.

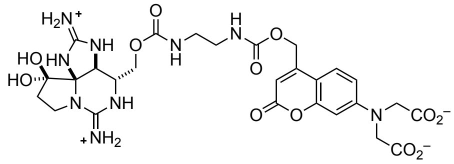

(7-[Bis(carboxymethyl)-amino]coumarin-4-yl)methyl N21-ethylcarbamate saxitoxin (STX-eac, 5). This compound was prepared in an analogous manner to **2** starting from STX-ea **1** (3.54 µmol) and **12** (3.3 mg, 7.07 µmol, 2.0 equiv). At a flow rate of 4 mL/min (gradient flow of 0→30% CH_3CN in 10 mM aqueous heptafluorobutyric acid over 60 min, 214 nm UV detection), **5** had a retention time of 44–46 min and was isolated as a white powder following lyophilization (2.17 µmol, 61%, ^{1}H NMR quantitation). This material was diluted in 9:1 10 mM aqueous CF_3CO_2H/CH_3CN solution and re-lyophilized after quantitation in order to exchange the heptafluorobutyrate counterions for trifluoroacetate counterions.

^{1}H NMR (600 MHz, D_2O): δ 7.61 (d, *J* = 8.9 Hz, 1H), 6.69 (dd, *J* = 8.9, 2.6 Hz, 1H), 6.58 (d, *J* = 2.6 Hz, 1H), 6.22 (s, 1H), 5.40–5.26 (m, 2H), 4.60 (s, 1H), 4.32 (s, 4H), 4.25 (t, *J* = 10.5 Hz, 1H), 3.91 (dd, *J* = 11.6, 5.3 Hz, 1H), 3.71 (t, *J* = 10.0 Hz, 1H), 3.59–3.53 (m, 1H), 3.51–3.35 (m, 2H), 3.32–3.15 (m, 3H), 2.42–2.24 (m, 2H) ppm; HRMS (ESI^{+}): $[MH]^{+}$ calcd. for $C_{27}H_{33}N_9O_{12}$, 676.2322; found, 676.2312.

6-Bromo-7-methoxycoumarin-4-yl)methoxycarbonyl-N-oxosuccinimide (9)

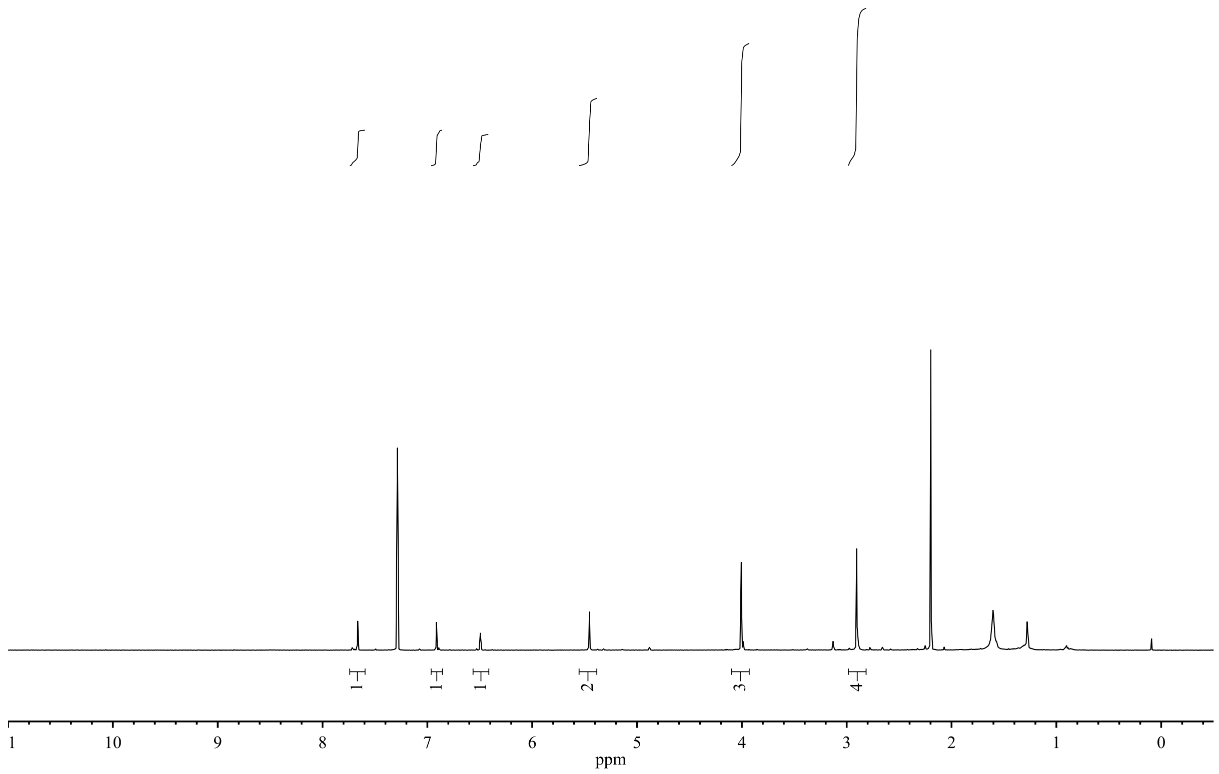

6-Bromo-7-hydroxycoumarin-4-yl)methoxycarbonyl-N-oxosuccinimide (10)

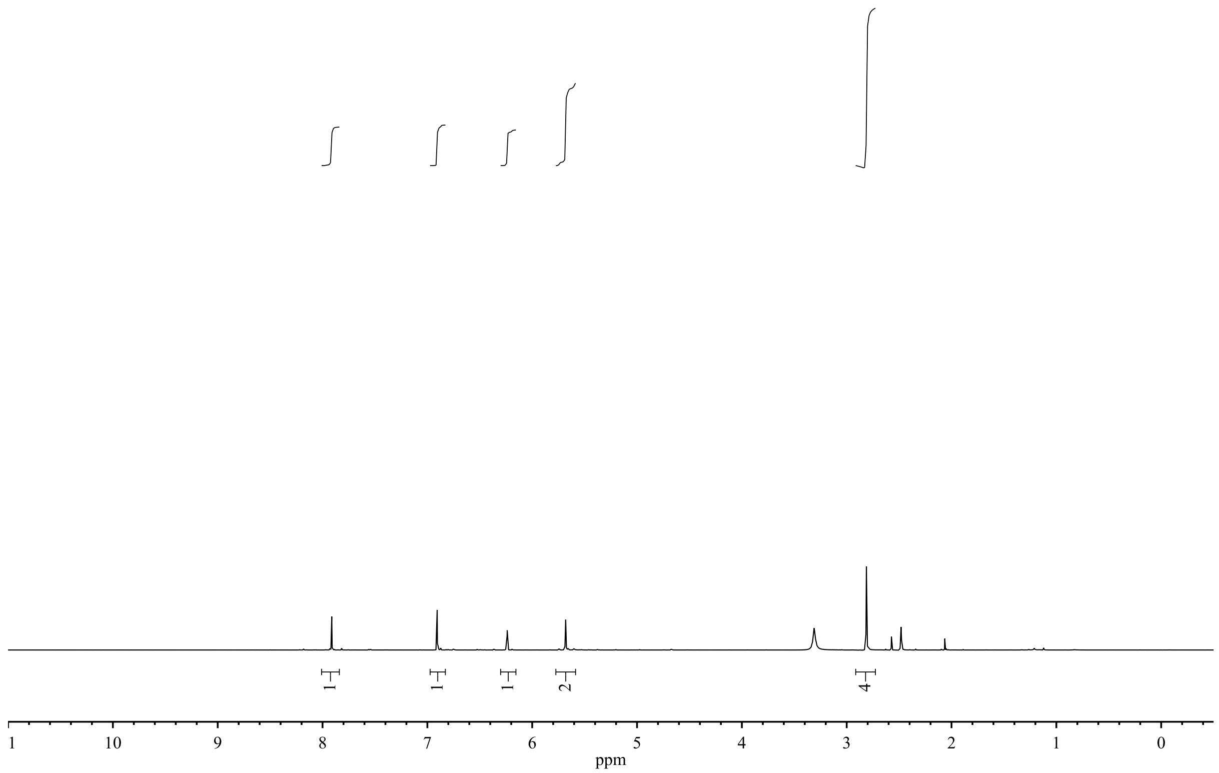

7-[Bis(tert-butoxycarbonylmethyl)-amino]coumarin-4-yl)methoxycarbonyl-N-oxosuccinimide (11)

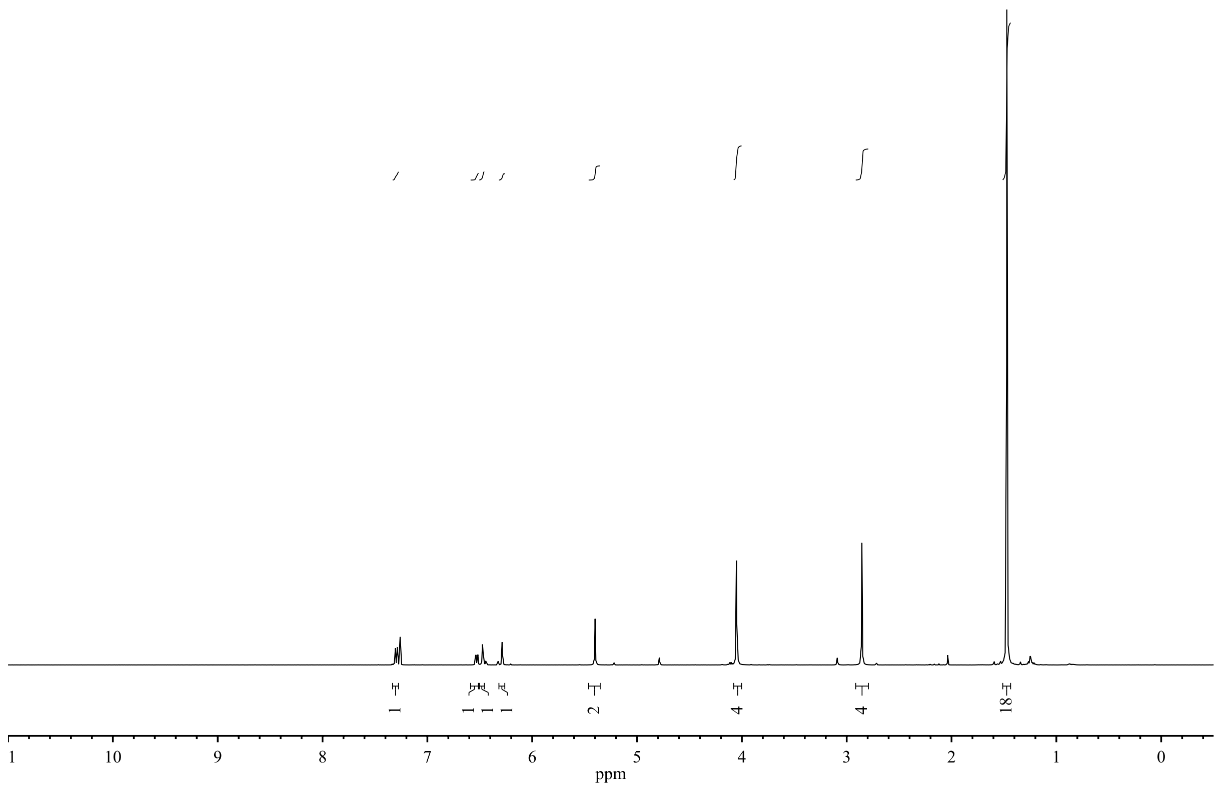

7-[Bis(carboxymethyl)-amino]coumarin-4-yl)methoxycarbonyl-N-oxosuccinimide (12)

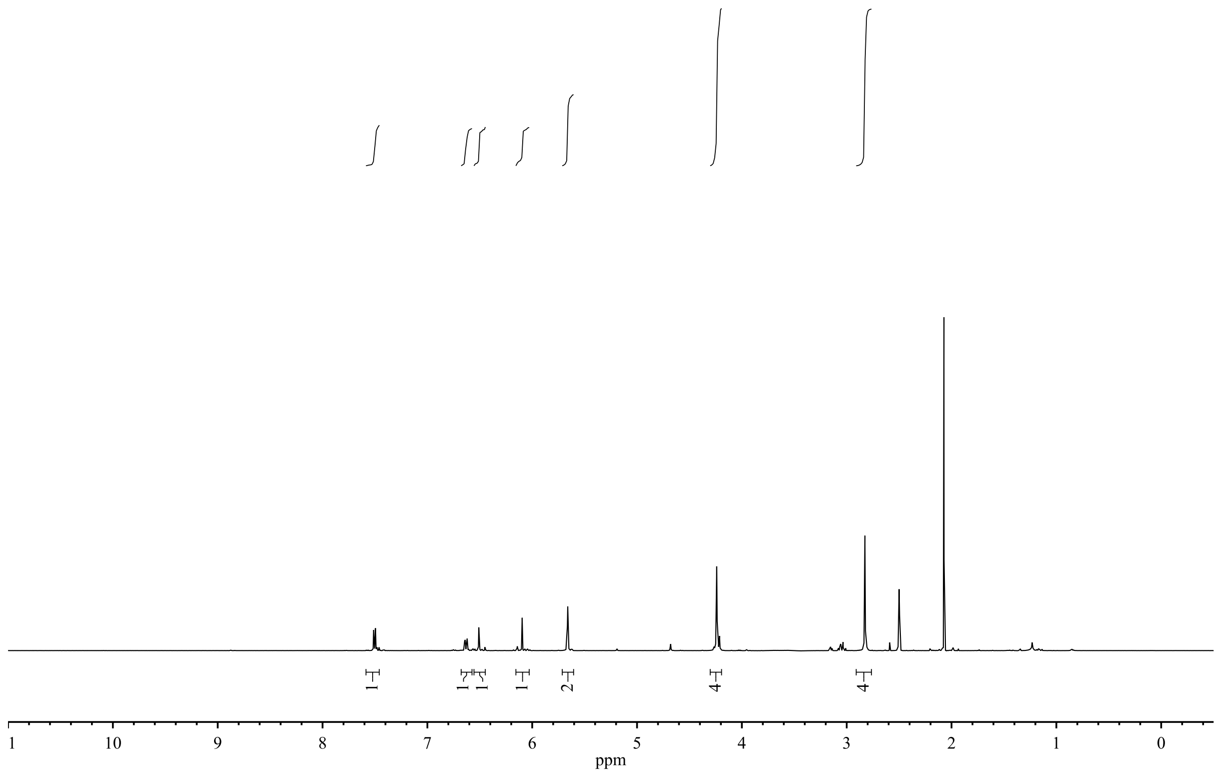

6-Bromo-7-methoxycoumarin-4-ylmethyl N21-ethylcarbamate-saxitoxin (2)

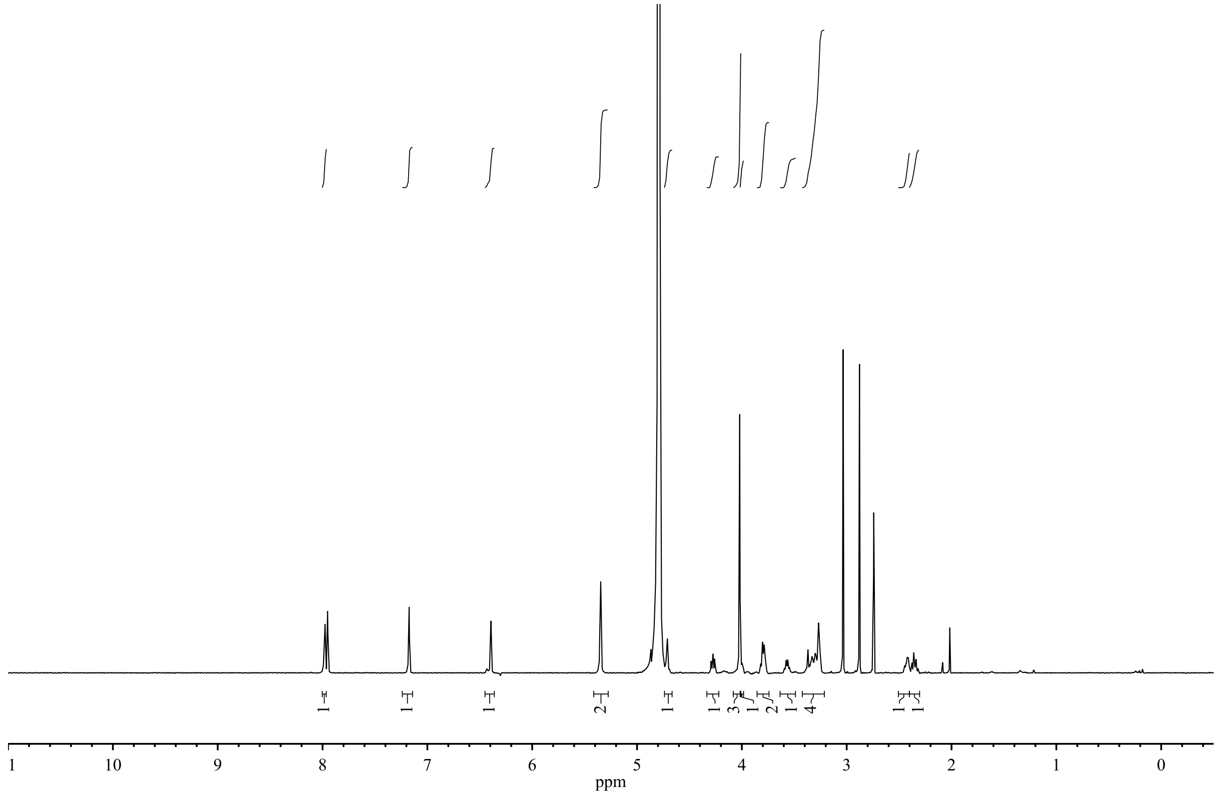

6-Bromo-7-hydroxycoumarin-4-ylmethyl N21-ethylcarbamate-saxitoxin (3)

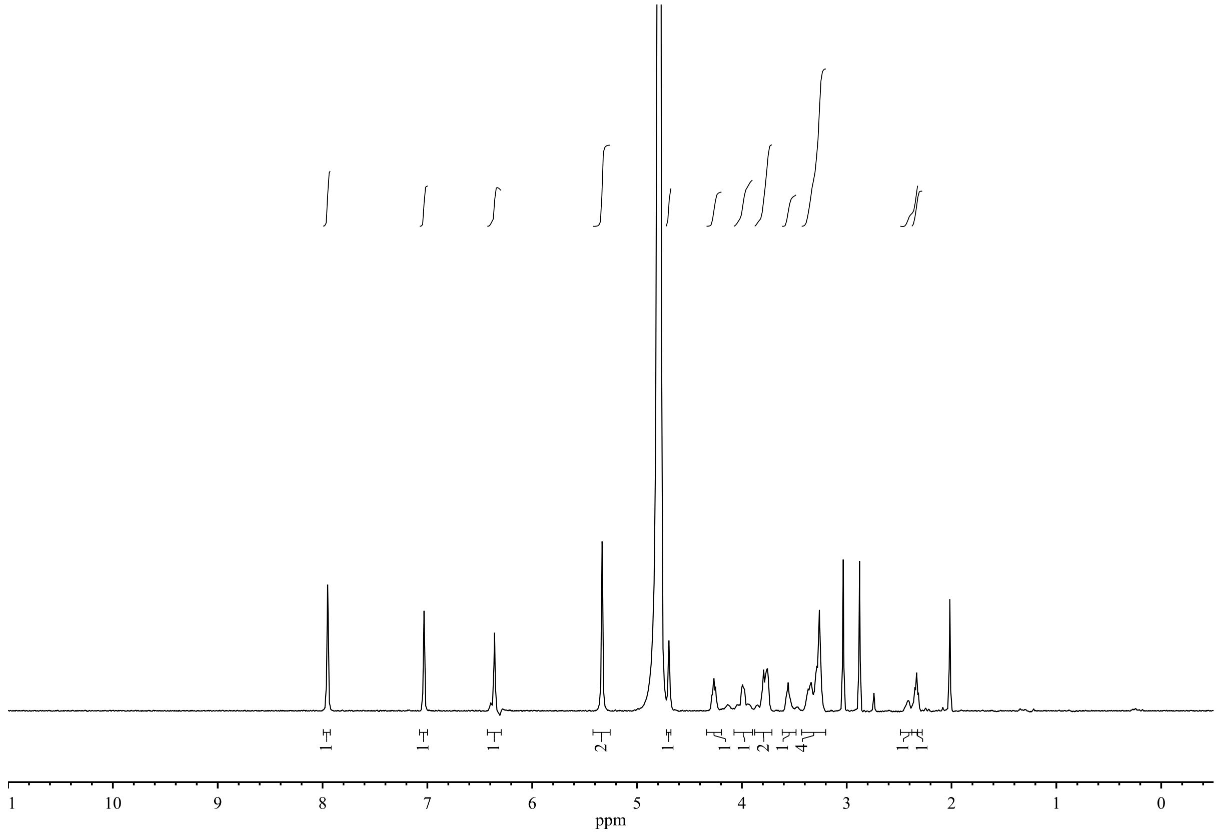

7-[Bis(tert-butoxycarbonylmethyl)-amino]coumarin-4-yl)methyl N21-ethylcarbamate-saxitoxin (4)

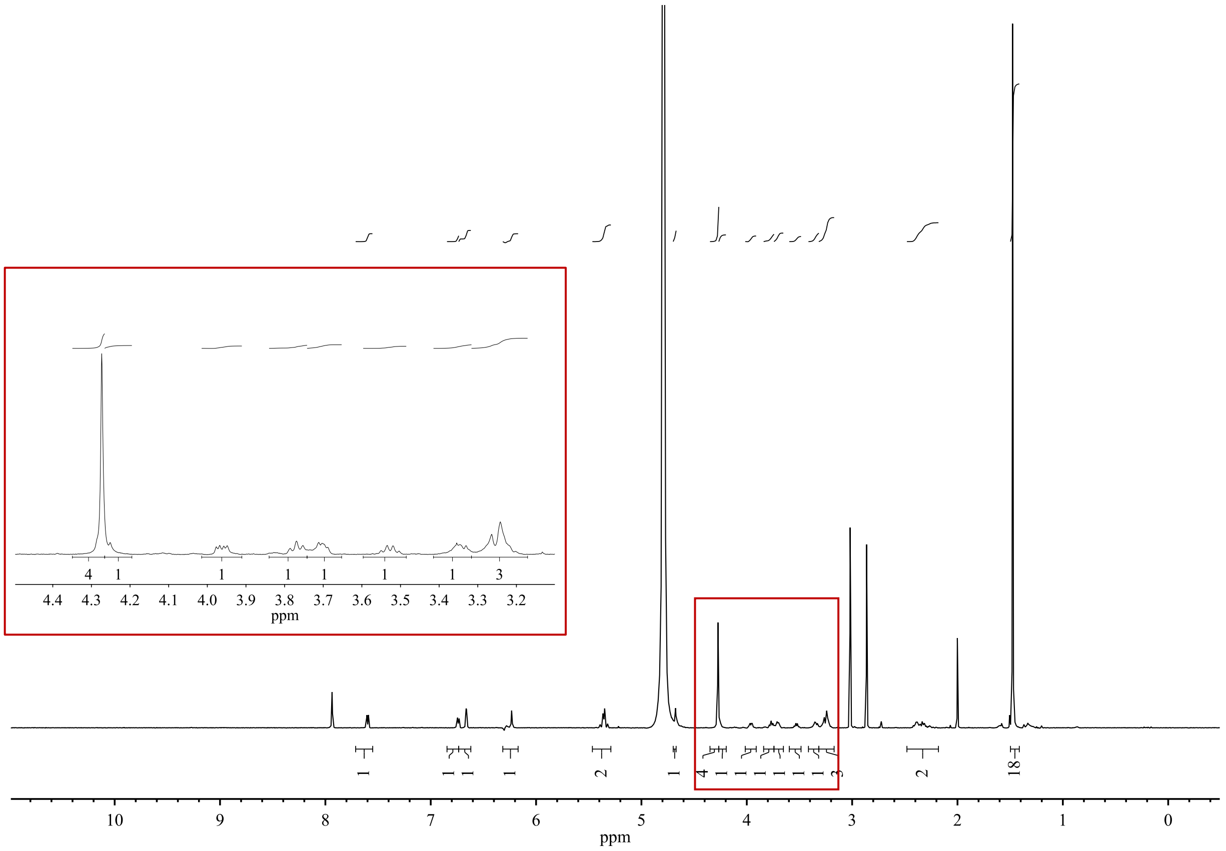

7-[Bis(carboxymethyl)-amino]coumarin-4-yl)methyl N21-ethylcarbamate-saxitoxin (5)

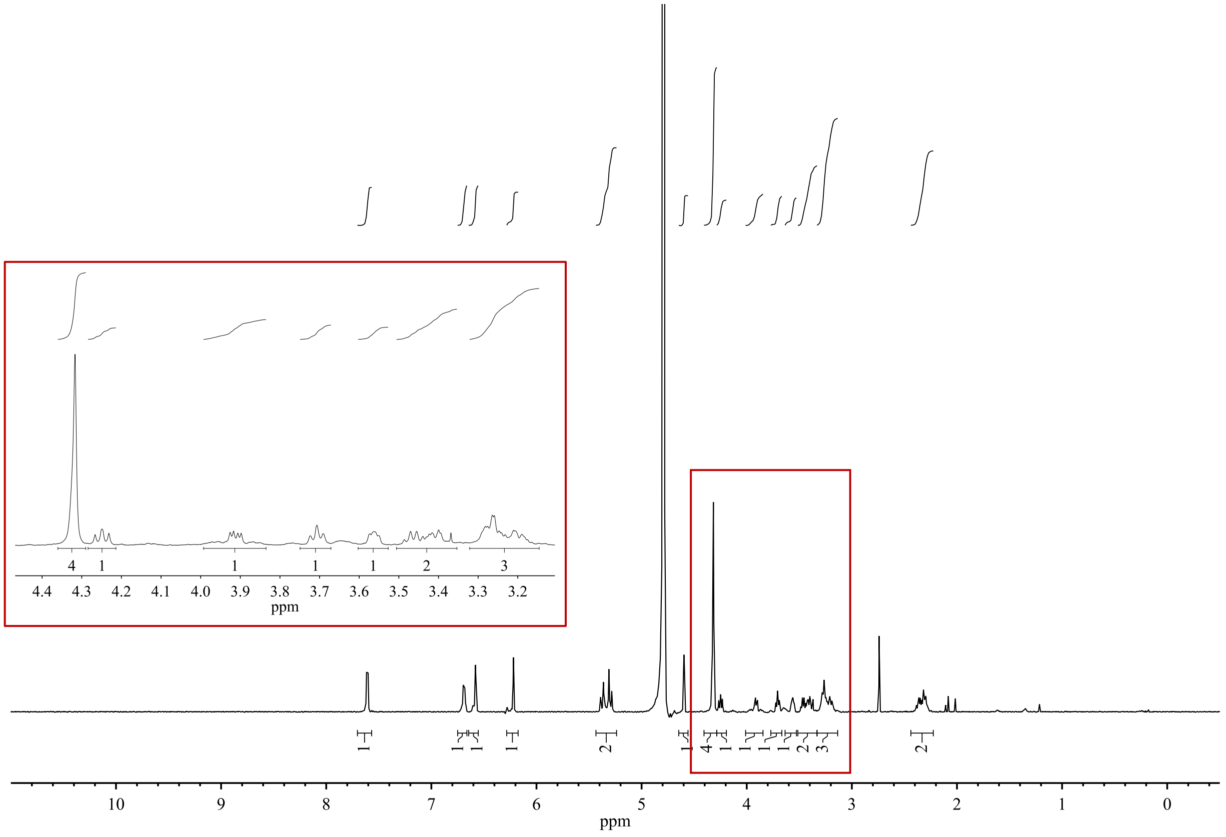

Supplementary References:

1. Hahn, R. & Strichartz, G. Effects of deuterium oxide on the rate and dissociation constants for saxitoxin and tetrodotoxin action. *J. Gen. Physiol*. **78**, 113–139 (1981).
