# Supplementary material for: Precise spatiotemporal control of voltage-gated sodium channels by photocaged saxitoxin
Source: Nat Commun. 2021 Jul 7;12:4171. doi: 10.1038/s41467-021-24392-2 (PMC8263607; doi:10.1038/s41467-021-24392-2)
Supplement: Supplementary file 2 — Reporting Summary [file 41467_2021_24392_MOESM2_ESM.pdf]

nature research

Corresponding author(s): Justin Du Bois

Last updated by author(s): 2021/5/14

Reporting Summary

Nature Research wishes to improve the reproducibility of the work that we publish. This form provides structure for consistency and transparency in reporting. For further information on Nature Research policies, see our Editorial Policies and the Editorial Policy Checklist.

Statistics

For all statistical analyses, confirm that the following items are present in the figure legend, table legend, main text, or Methods section.

n/a	Confirmed	
☐	☒	The exact sample size (*n*) for each experimental group/condition, given as a discrete number and unit of measurement
☐	☒	A statement on whether measurements were taken from distinct samples or whether the same sample was measured repeatedly
☐	☒	The statistical test(s) used AND whether they are one- or two-sided<br>*Only common tests should be described solely by name; describe more complex techniques in the Methods section.*
☐	☒	A description of all covariates tested
☐	☒	A description of any assumptions or corrections, such as tests of normality and adjustment for multiple comparisons
☐	☒	A full description of the statistical parameters including central tendency (e.g. means) or other basic estimates (e.g. regression coefficient) AND variation (e.g. standard deviation) or associated estimates of uncertainty (e.g. confidence intervals)
☐	☒	For null hypothesis testing, the test statistic (e.g. *F*, *t*, *r*) with confidence intervals, effect sizes, degrees of freedom and *P* value noted<br>*Give P values as exact values whenever suitable.*
☒	☐	For Bayesian analysis, information on the choice of priors and Markov chain Monte Carlo settings
☒	☐	For hierarchical and complex designs, identification of the appropriate level for tests and full reporting of outcomes
☒	☐	Estimates of effect sizes (e.g. Cohen's *d*, Pearson's *r*), indicating how they were calculated

Our web collection on statistics for biologists contains articles on many of the points above.

Software and code

Policy information about availability of computer code

Data collection	To acquire CHO-K1, Nav1.2 CHO, and rat hippocampal data we used pCLAMP software from Molecular Devices (Clampex 10.4 and 11.1). To acquire callosal data we used OpenDeveloper from TDT (Tucker-Davis Technologies, Inc); we then used TDT2mat.m code available on TDT (Tucker-Davis Technologies, Inc) to export on Matlab.
Data analysis	For CHO-K1, Nav1.2 CHO, and rat hippocampal experimental analysis, Graphpad Prism 8 was used as described in the manuscript. For callosal experiment analysis, a custom Matlab code was used to obtain the CSD (Current source density) from TDT recorded LFP data spacing = 0.1; the following formula was used:<br>csd=-diff(diff(data,1,chan),1,chan)./sp2;<br>(with data= lfp data obtained from TDT2mat.m extraction,chan= 2, sp2= spacing*spacing (probe spacing is 100um in our case so spacing=0.1)

For manuscripts utilizing custom algorithms or software that are central to the research but not yet described in published literature, software must be made available to editors and reviewers. We strongly encourage code deposition in a community repository (e.g. GitHub). See the Nature Research guidelines for submitting code & software for further information.

Data

Policy information about availability of data

All manuscripts must include a data availability statement. This statement should provide the following information, where applicable:

- Accession codes, unique identifiers, or web links for publicly available datasets
- A list of figures that have associated raw data
- A description of any restrictions on data availability

The datasets generated during the current study are available form the corresponding author on reasonable request. All synthetic characterization data generated

during this study are included in the supplementary information files. Source data are provided with this paper.

Field-specific reporting

Please select the one below that is the best fit for your research. If you are not sure, read the appropriate sections before making your selection.

☒ Life sciences ☐ Behavioural & social sciences ☐ Ecological, evolutionary & environmental sciences

For a reference copy of the document with all sections, see nature.com/documents/nr-reporting-summary-flat.pdf

Life sciences study design

All studies must disclose on these points even when the disclosure is negative.

Sample size	No sample size calculation was performed. For data collected on dissociated cells, sample sizes were chosen based on prior experiments with neurotoxins within the lab. Measurements on five or more cells typically yielded SEMs of less than 5%, consistent with literature precedent. For slice experiments, at least three mice per group were used in order to assess reproducibility. Sample sizes were chosen based on established standards in the lab. At least eight brain slices were used in each condition, which is sufficient to reliably detect changes of the magnitudes shown in the paper, i.e. 10–50% saxitoxin dependent reduction.
Data exclusions	For data collected on dissociated cells, cells were chosen as described in the Methods section of the manuscript. For voltage-clamp data on dissociated cells, data was included if the cell had >1 nA of sodium current and the leak current remained less than –0.3 nA. These cutoffs were predetermined, are consistent with literature precedent, and ensure that (a) the signal:noise ratio is low and (b) cell quality is maintained throughout the experiment.<br><br>Specifically for IC50 data, cells that recovered current to within +/-10% after initial toxin application and wash-off were used. These exclusion criteria were also pre-established—the rationale being that IC50 data should reflect the effects of the applied toxin and not inherent changes in the sodium current of a cell.<br><br>For current-clamp experiments, all cells firing action potential trains with frequencies greater than 5 Hz were taken, provided a seal was maintained for the duration of the experiment. Cells were chosen in this way to ensure precise reporting of action potential block.<br><br>Slice experiments were conducted as described in the manuscript; when measurements were not possible data was included in a failure graph.
Replication	For data collected on dissociated cells, all results were replicated against at least three distinct cells (most often five or more). Data were reproducible (see error measurements in the manuscript and supporting information). For slice experiments, each condition was tested on a least three mice with two slices taken per mouse. We confirmed reproducibility by showing that effects were consistently found in at least ten slices total from at least five mice. See manuscript and supporting information for exact replicate numbers and associated error measurements.
Randomization	Allocation was random.
Blinding	Blinding was not possible due to the requisite presence or absence of saxitoxin photocage in the test and control groups, respectively. Likewise, laser application requires manual input and cannot be blinded.

Reporting for specific materials, systems and methods

We require information from authors about some types of materials, experimental systems and methods used in many studies. Here, indicate whether each material, system or method listed is relevant to your study. If you are not sure if a list item applies to your research, read the appropriate section before selecting a response.

Materials & experimental systems

n/a	Involved in the study	
☒	☐	Antibodies
☐	☒	Eukaryotic cell lines
☒	☐	Palaeontology and archaeology
☐	☒	Animals and other organisms
☒	☐	Human research participants
☒	☐	Clinical data
☒	☐	Dual use research of concern

Methods

n/a	Involved in the study	
☒	☐	ChIP-seq
☒	☐	Flow cytometry
☒	☐	MRI-based neuroimaging

Eukaryotic cell lines

Policy information about cell lines

Cell line source(s)	CHO-K1 cells: ATCC CCL-61<br>Nav1.2 CHO cells: Dr. William A. Catterall, see: West, J. W. et al. Efficient expression of rat brain type IIA Na+ channel alpha subunits in a somatic cell line. Neuron 8, 59–70 (1992).
Authentication	No authentication was performed. Cells were used at low passage numbers from initial receipt (p < 20).
Mycoplasma contamination	CHO-K1 cells tested negative for mycoplasma contamination; Nav1.2 CHO cells were not tested.
Commonly misidentified lines<br>(See ICLAC register)	No commonly misidentified cell lines were used in this study.

Animals and other organisms

Policy information about studies involving animals; ARRIVE guidelines recommended for reporting animal research

Laboratory animals	Male and female E18 Sprague Dawley rats from Charles River were used for experiments with dissociated hippocampal neurons. Young (~P35) male and female wild type mice from the C3HeB/FeJ-Scn8amed/J strain were used for slice experiments. Mice were housed at a density of five per cage under a light/dark cycle of 7 am – 7 pm at ambient temperature (20 °C) and humidity.
Wild animals	No wild animals were used in the study.
Field-collected samples	No field collected samples were used in the study.
Ethics oversight	Stanford administrative panel on laboratory animal care (APLAC). Protocols: Modulating electrical activity in primary neurons; Neurophysiology experimental epilepsy.

Note that full information on the approval of the study protocol must also be provided in the manuscript.
